# Supplementary figures and images for: Endophilin A1 facilitates organization of the GABAergic postsynaptic machinery to maintain excitation-inhibition balance
Source: eLife. 2025 Oct 2;13:RP102792. doi: 10.7554/eLife.102792 (PMC12490859; doi:10.7554/eLife.102792)

Figure 3E, Source Data

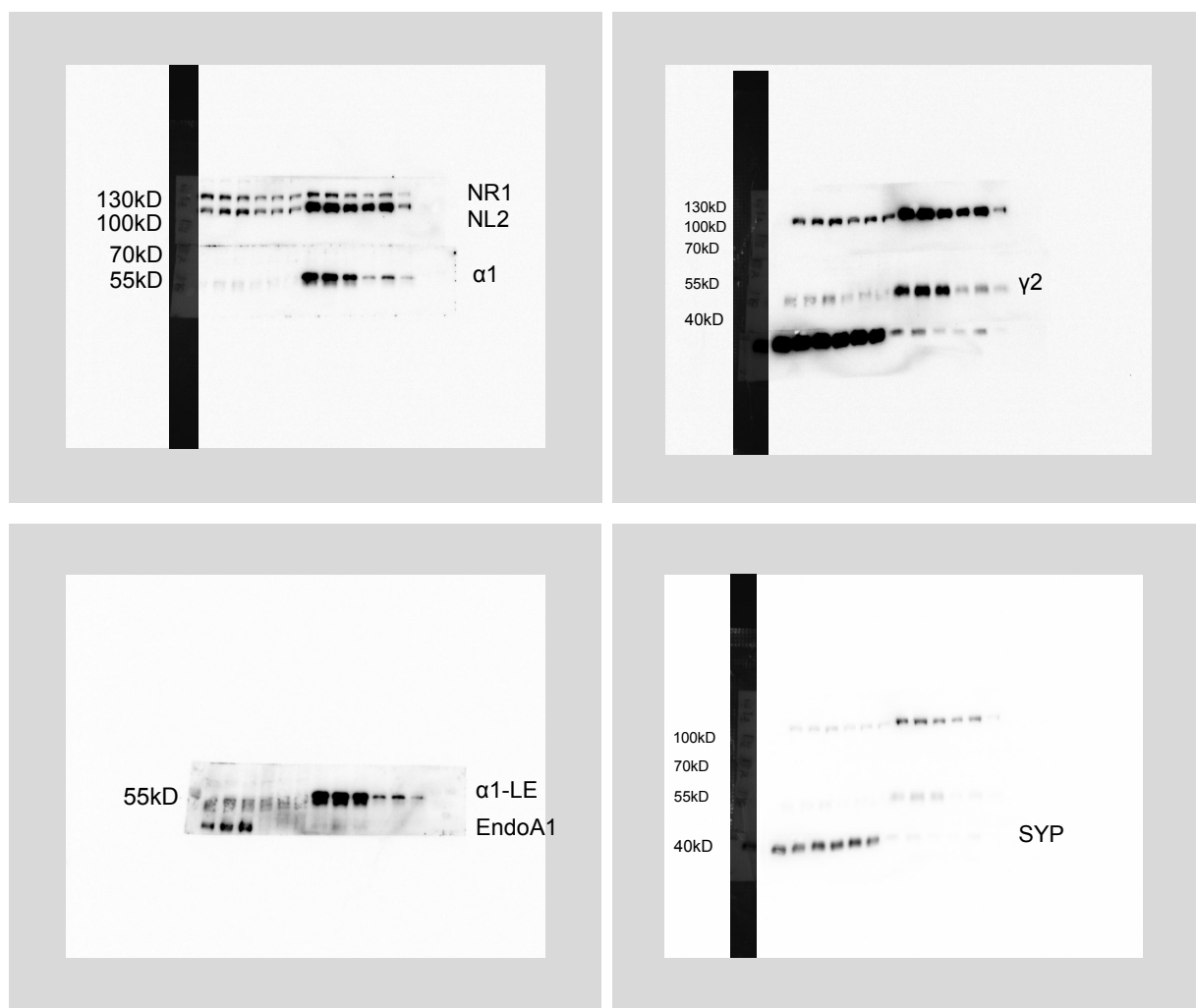

Supplement: Figure 3—source data 1. [file elife-102792-fig3-data1.zip › Figure 3-source data 1/Figure 3E-source data 1.pdf]

Figure 3J, Source Data

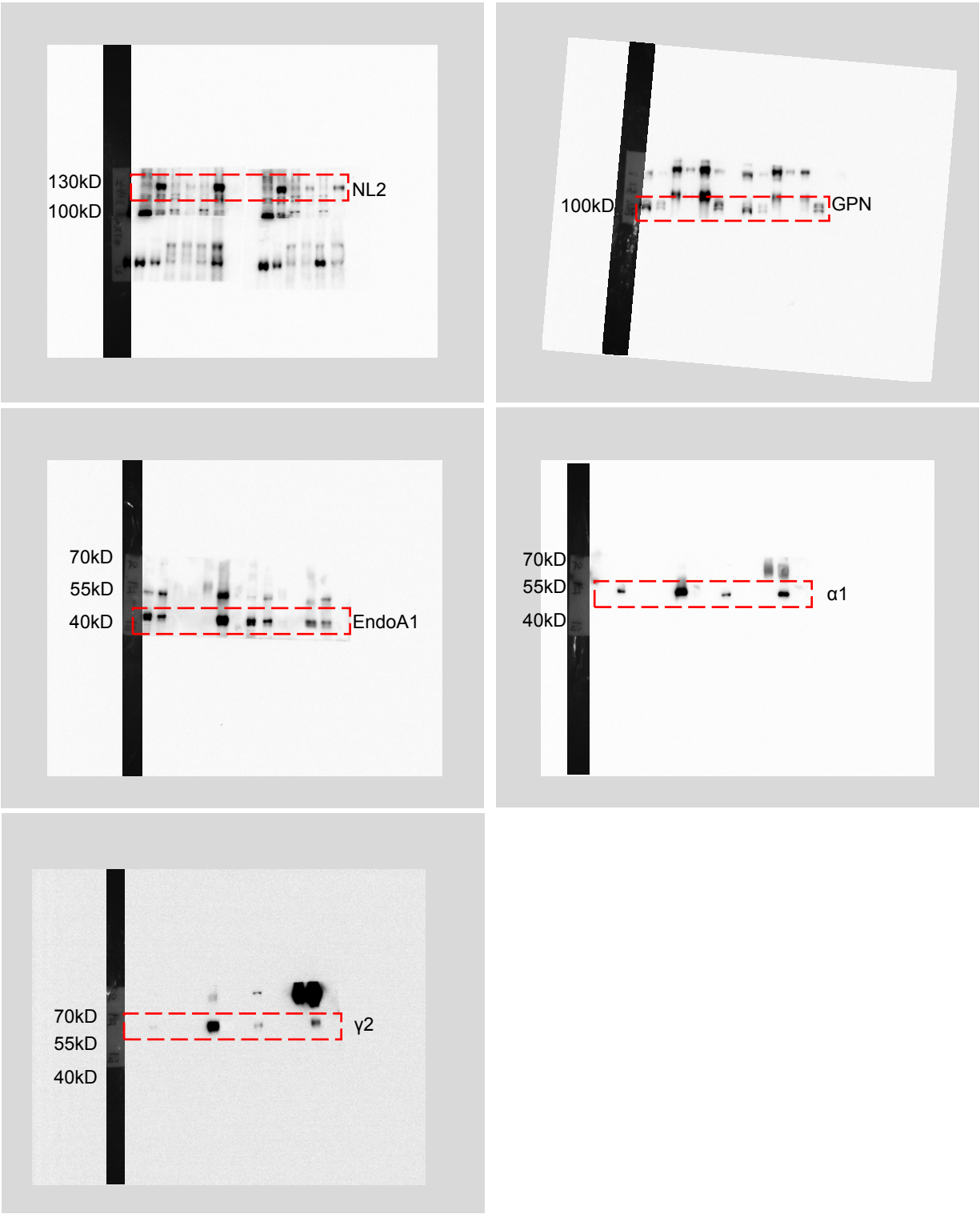

Supplement: Figure 3—source data 1. [file elife-102792-fig3-data1.zip › Figure 3-source data 1/Figure 3J-source data 1.pdf]

Figure 3K, Source Data

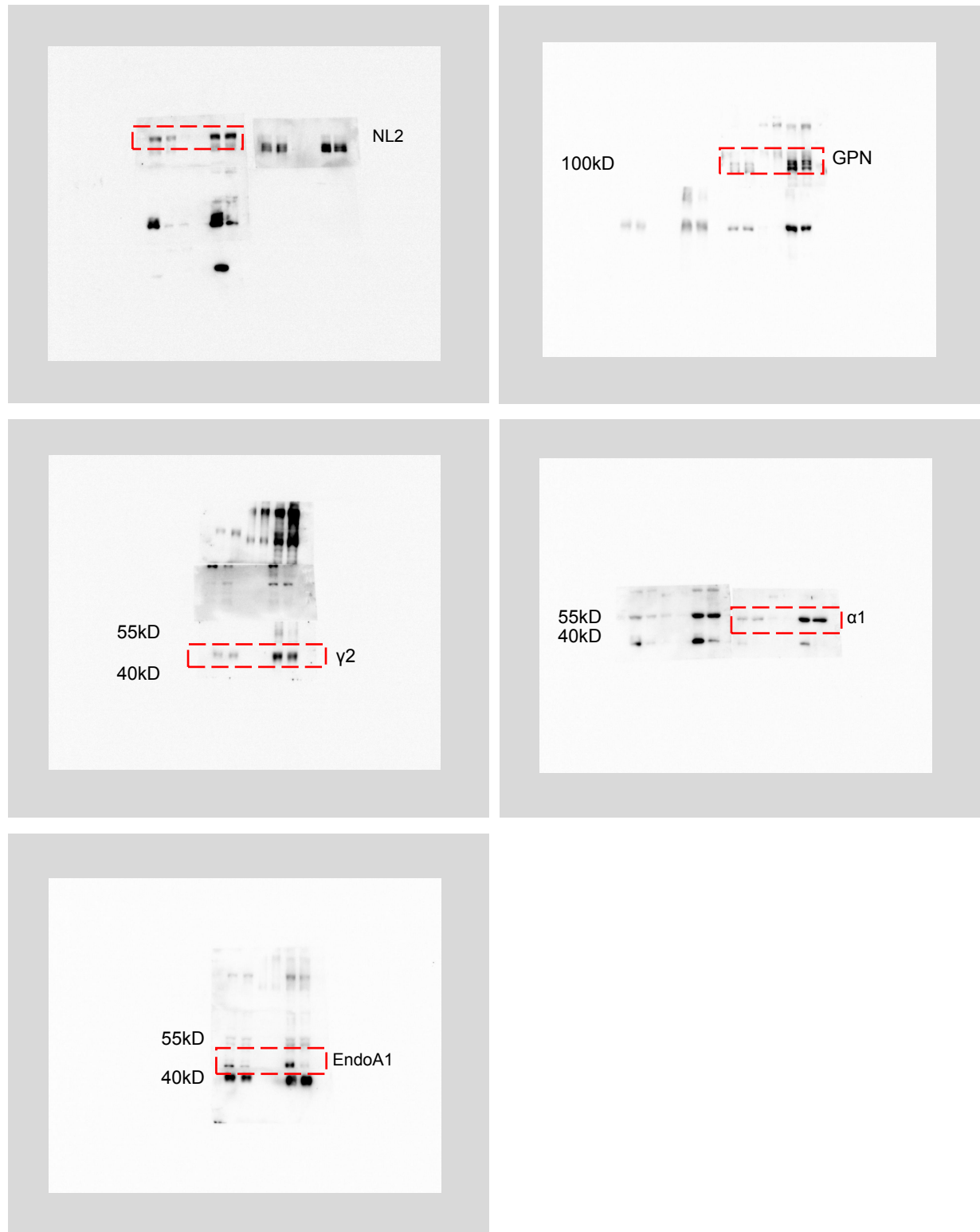

Supplement: Figure 3—source data 1. [file elife-102792-fig3-data1.zip › Figure 3-source data 1/Figure 3K-source data 1.pdf]

Figure 3L, Source Data

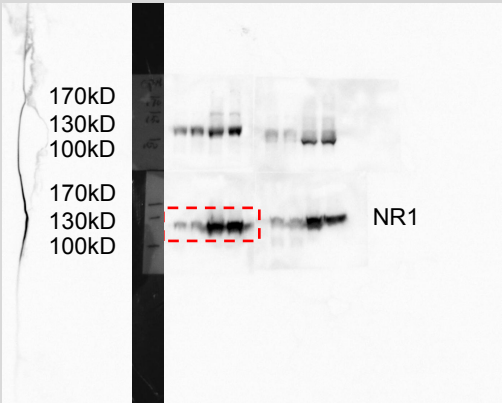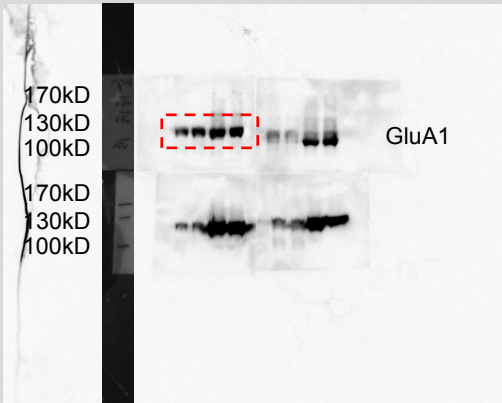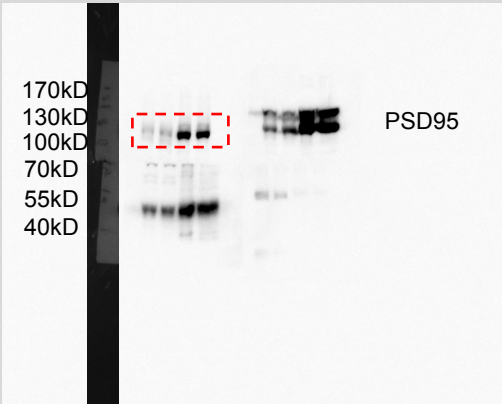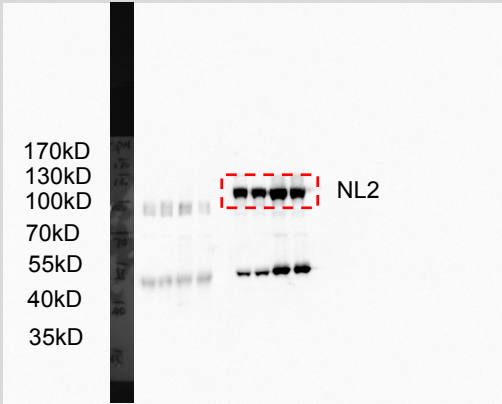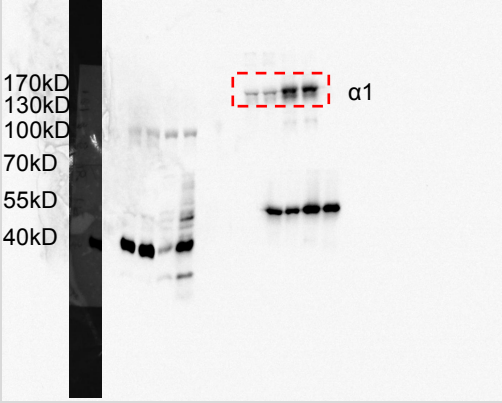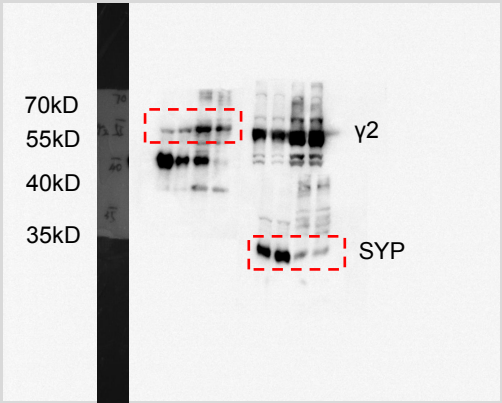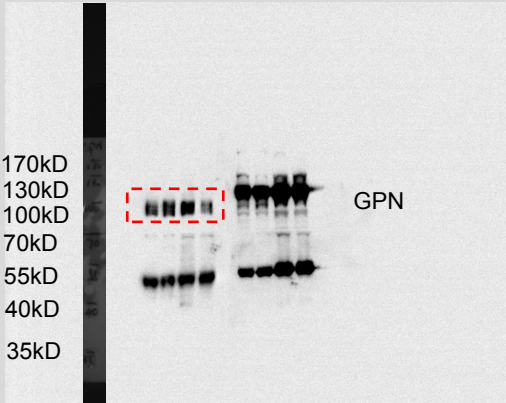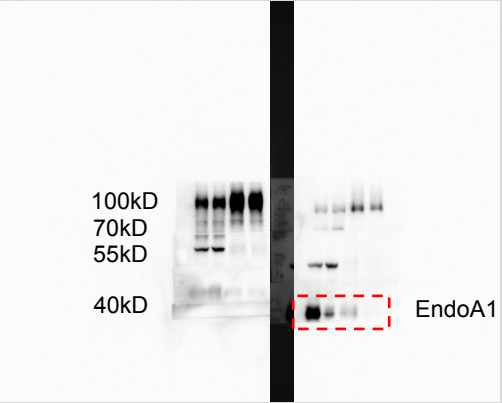

Supplement: Figure 3—source data 1. [file elife-102792-fig3-data1.zip › Figure 3-source data 1/Figure 3L-source data 1.pdf]

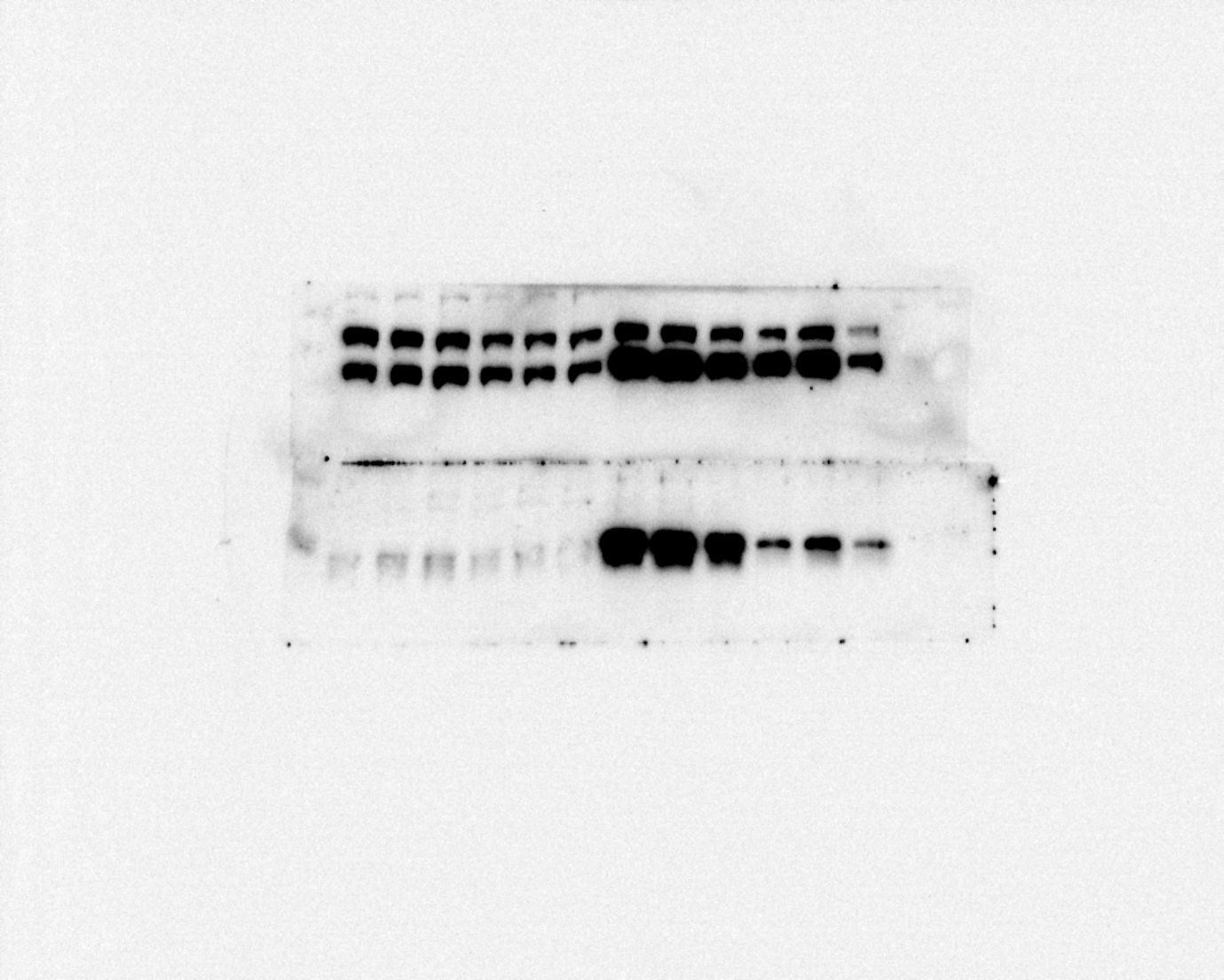

Supplement: Figure 3—source data 2. [file elife-102792-fig3-data2.zip › Figure 3-source data 2/Figure 3E_Source data 2/Figure 3E_Source data_anti alpha1-LE.tif]

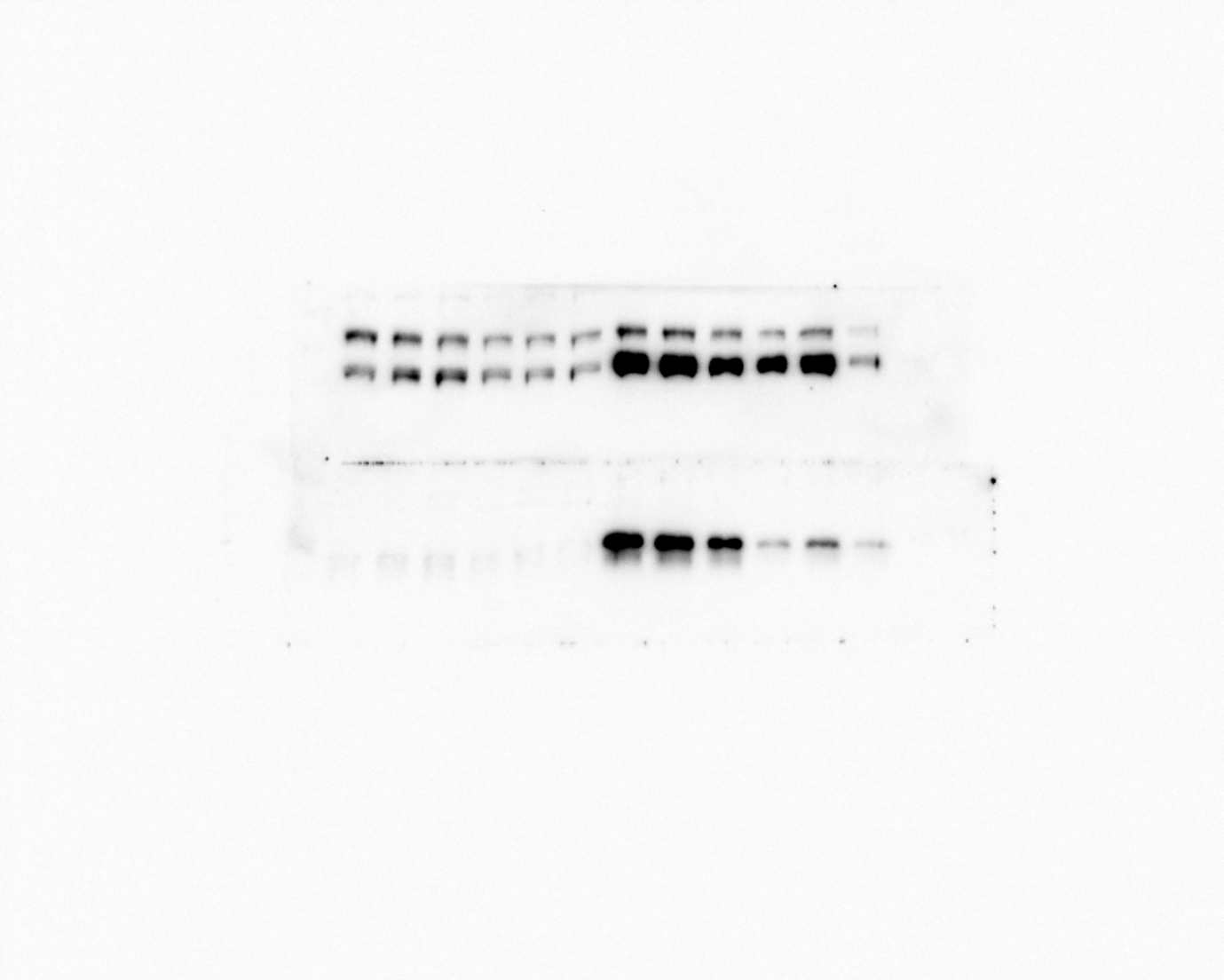

Supplement: Figure 3—source data 2. [file elife-102792-fig3-data2.zip › Figure 3-source data 2/Figure 3E_Source data 2/Figure 3E_Source data_anti alpha1.tif]

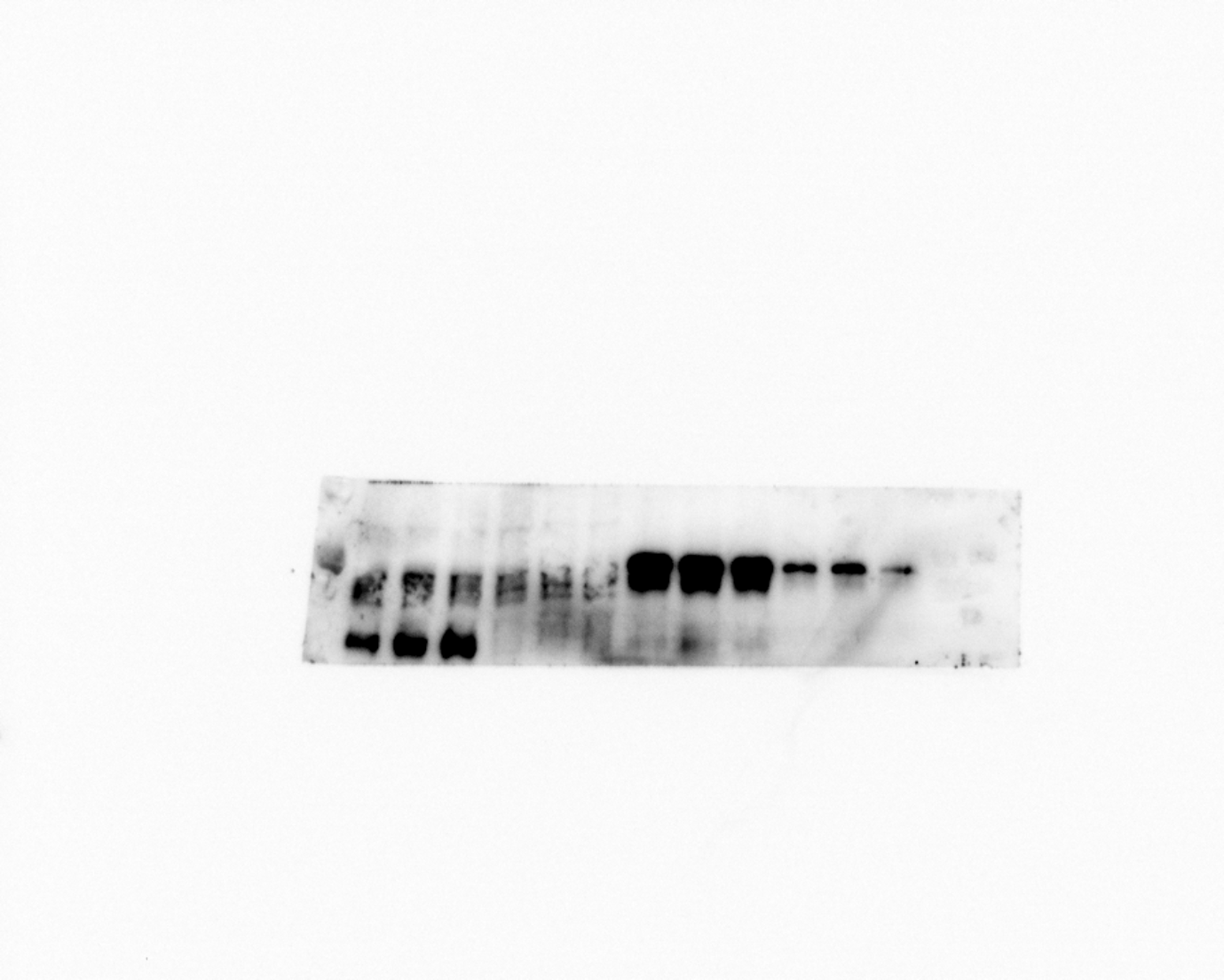

Supplement: Figure 3—source data 2. [file elife-102792-fig3-data2.zip › Figure 3-source data 2/Figure 3E_Source data 2/Figure 3E_Source data_anti EndoA1.tif]

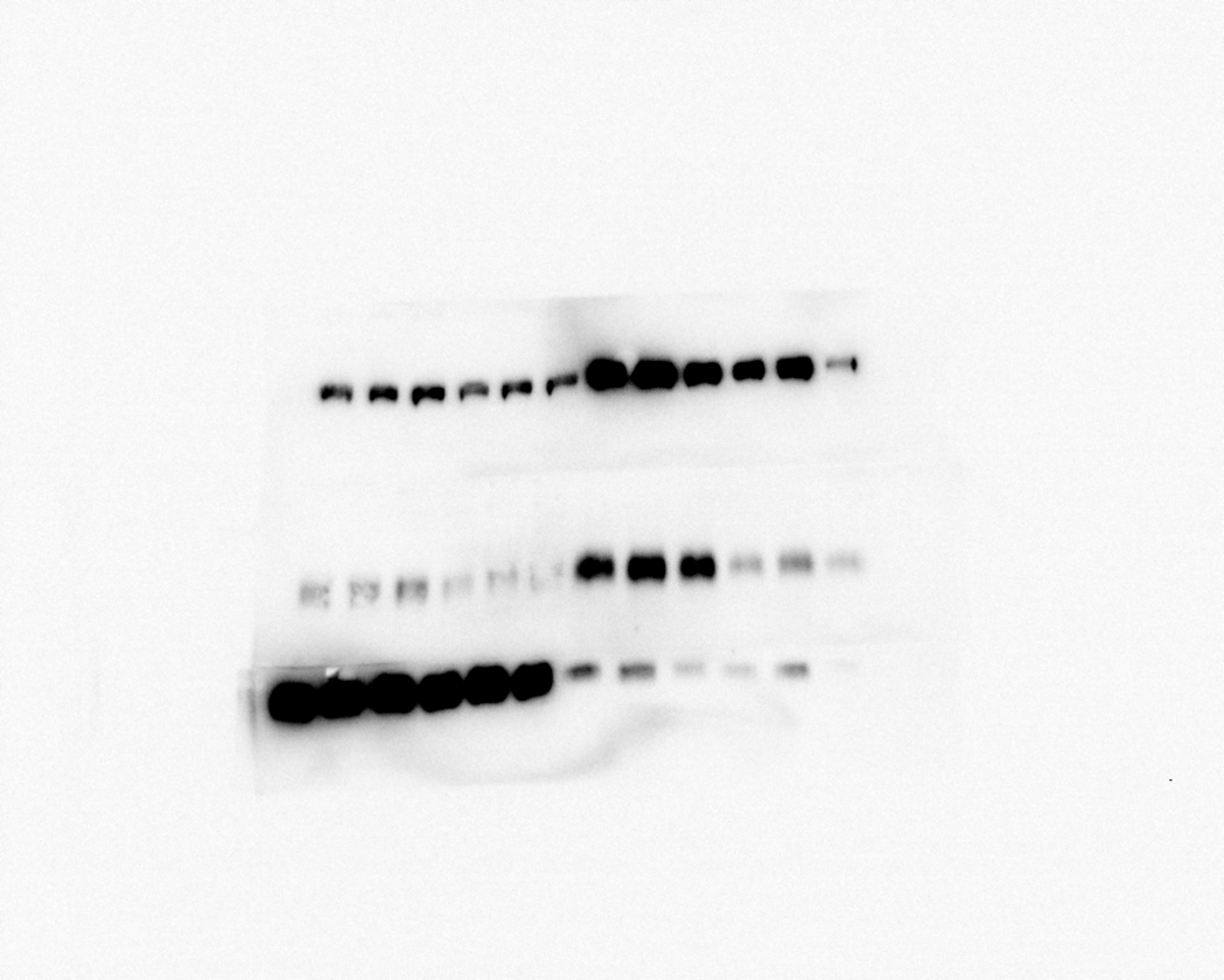

Supplement: Figure 3—source data 2. [file elife-102792-fig3-data2.zip › Figure 3-source data 2/Figure 3E_Source data 2/Figure 3E_Source data_anti gamma2.tif]

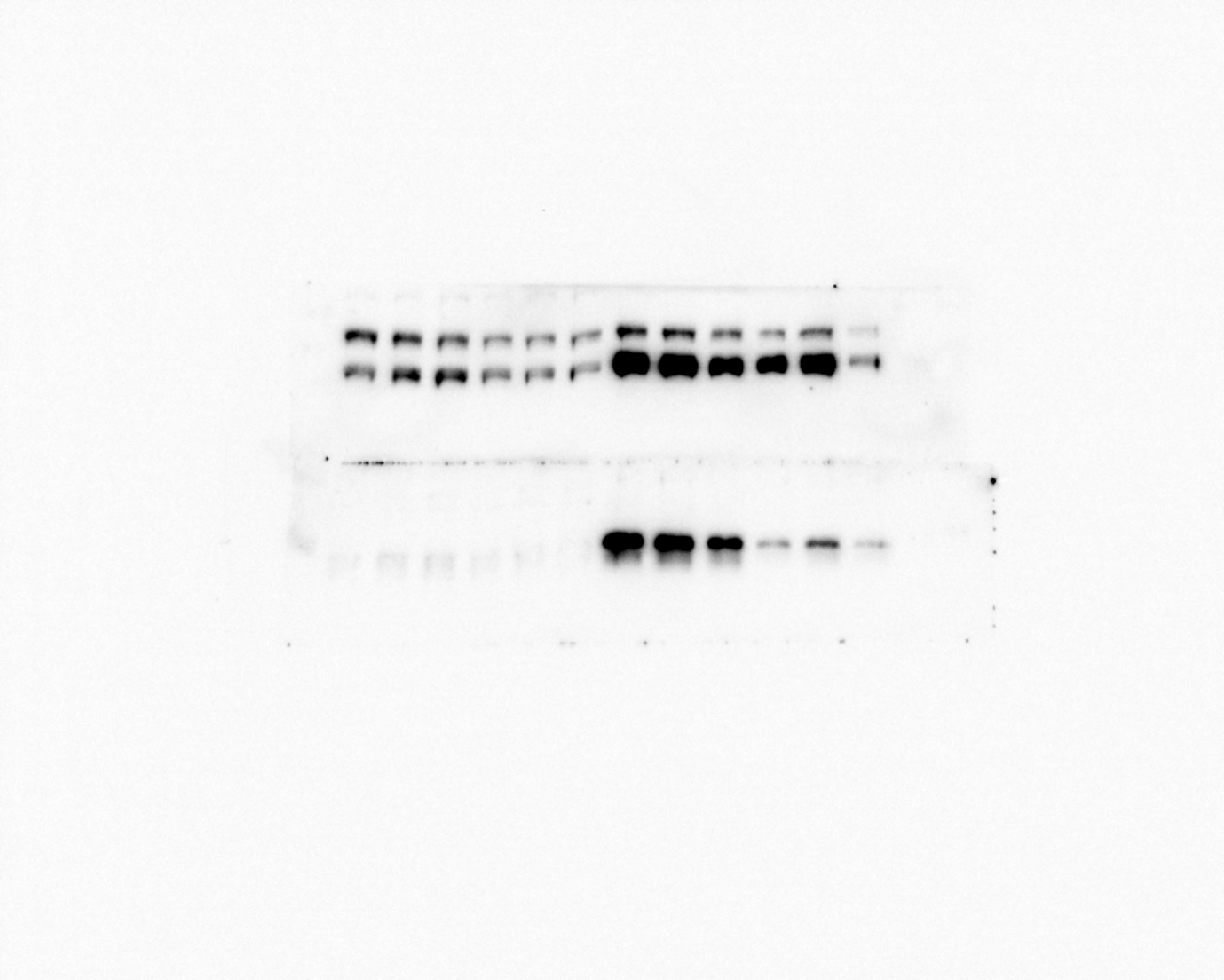

Supplement: Figure 3—source data 2. [file elife-102792-fig3-data2.zip › Figure 3-source data 2/Figure 3E_Source data 2/Figure 3E_Source data_anti NR1 and NL2.tif]

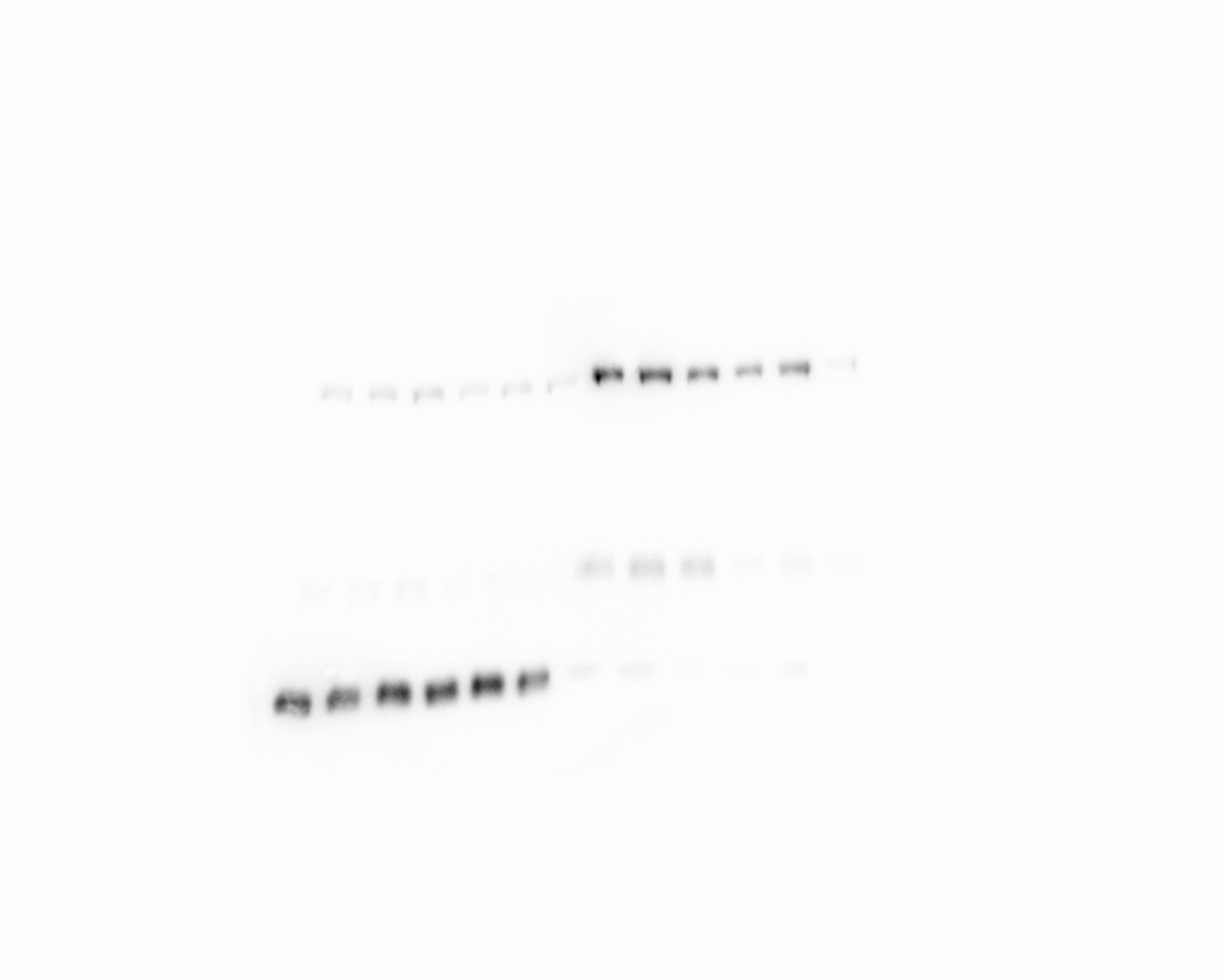

Supplement: Figure 3—source data 2. [file elife-102792-fig3-data2.zip › Figure 3-source data 2/Figure 3E_Source data 2/Figure 3E_Source data_anti SYP.tif]

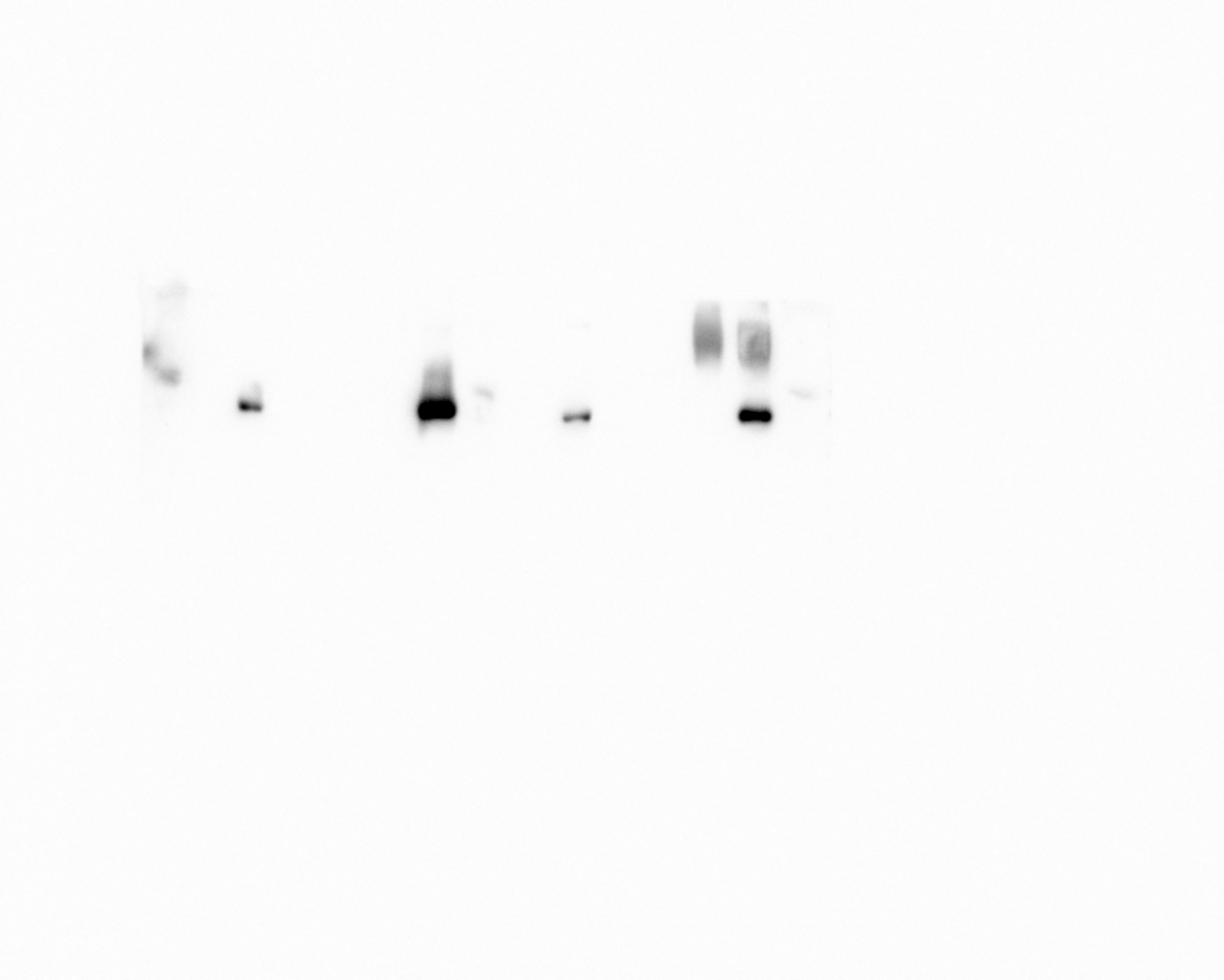

Supplement: Figure 3—source data 2. [file elife-102792-fig3-data2.zip › Figure 3-source data 2/Figure 3J_Source data 2/Figure 3J_Source data_anti alpha1.tif]

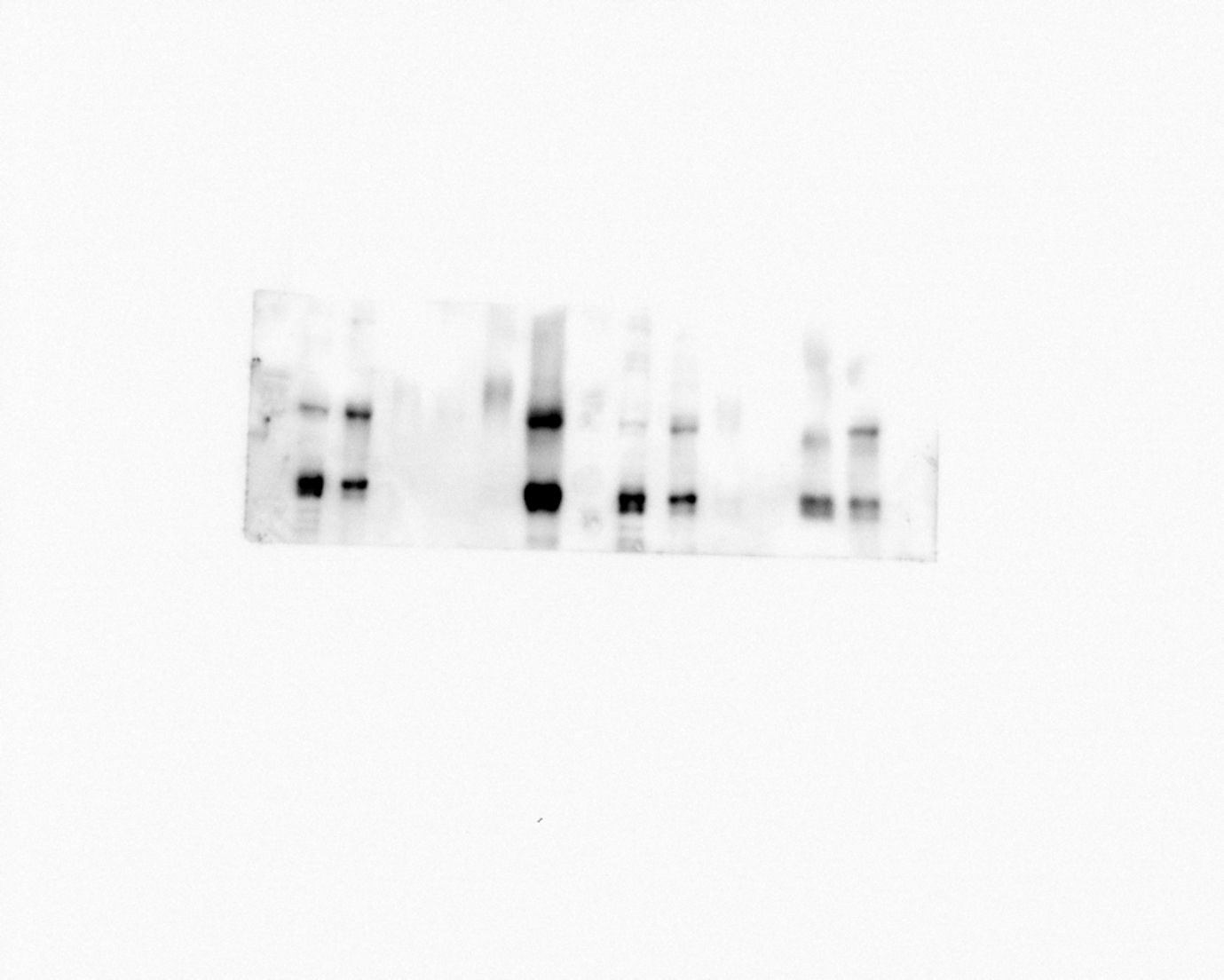

Supplement: Figure 3—source data 2. [file elife-102792-fig3-data2.zip › Figure 3-source data 2/Figure 3J_Source data 2/Figure 3J_Source data_anti EndoA1.tif]

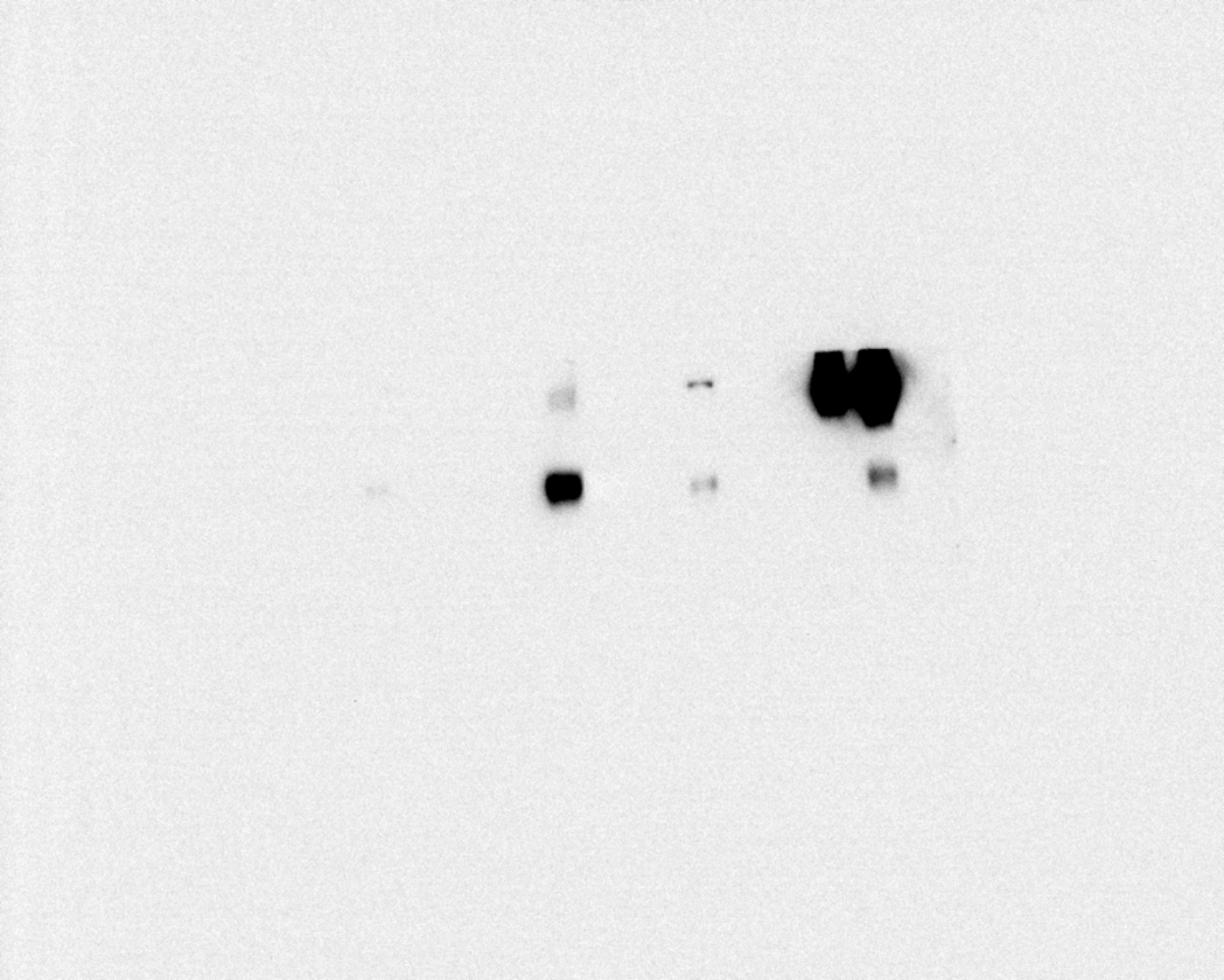

Supplement: Figure 3—source data 2. [file elife-102792-fig3-data2.zip › Figure 3-source data 2/Figure 3J_Source data 2/Figure 3J_Source data_anti gamma2.tif]

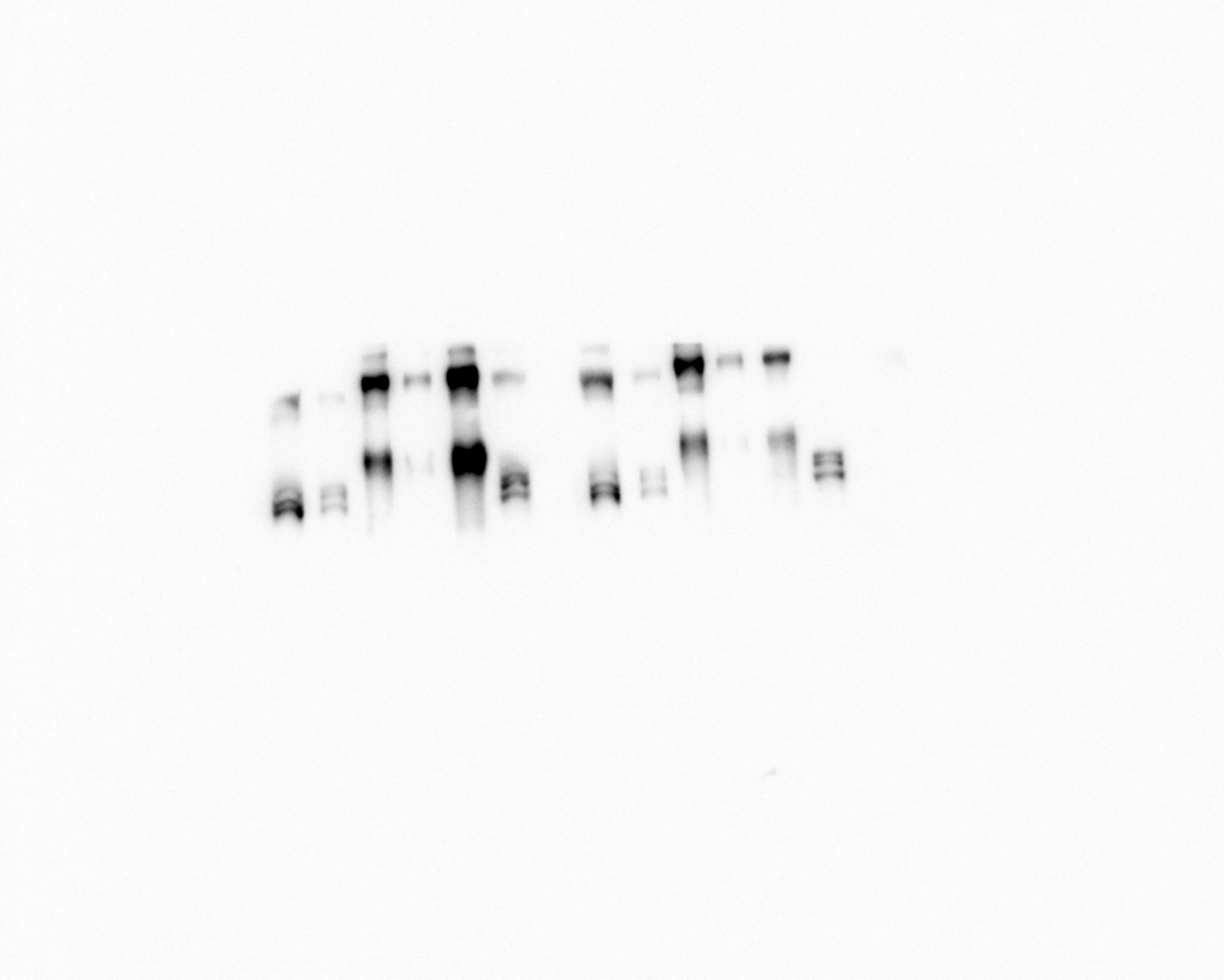

Supplement: Figure 3—source data 2. [file elife-102792-fig3-data2.zip › Figure 3-source data 2/Figure 3J_Source data 2/Figure 3J_Source data_anti GPN.tif]

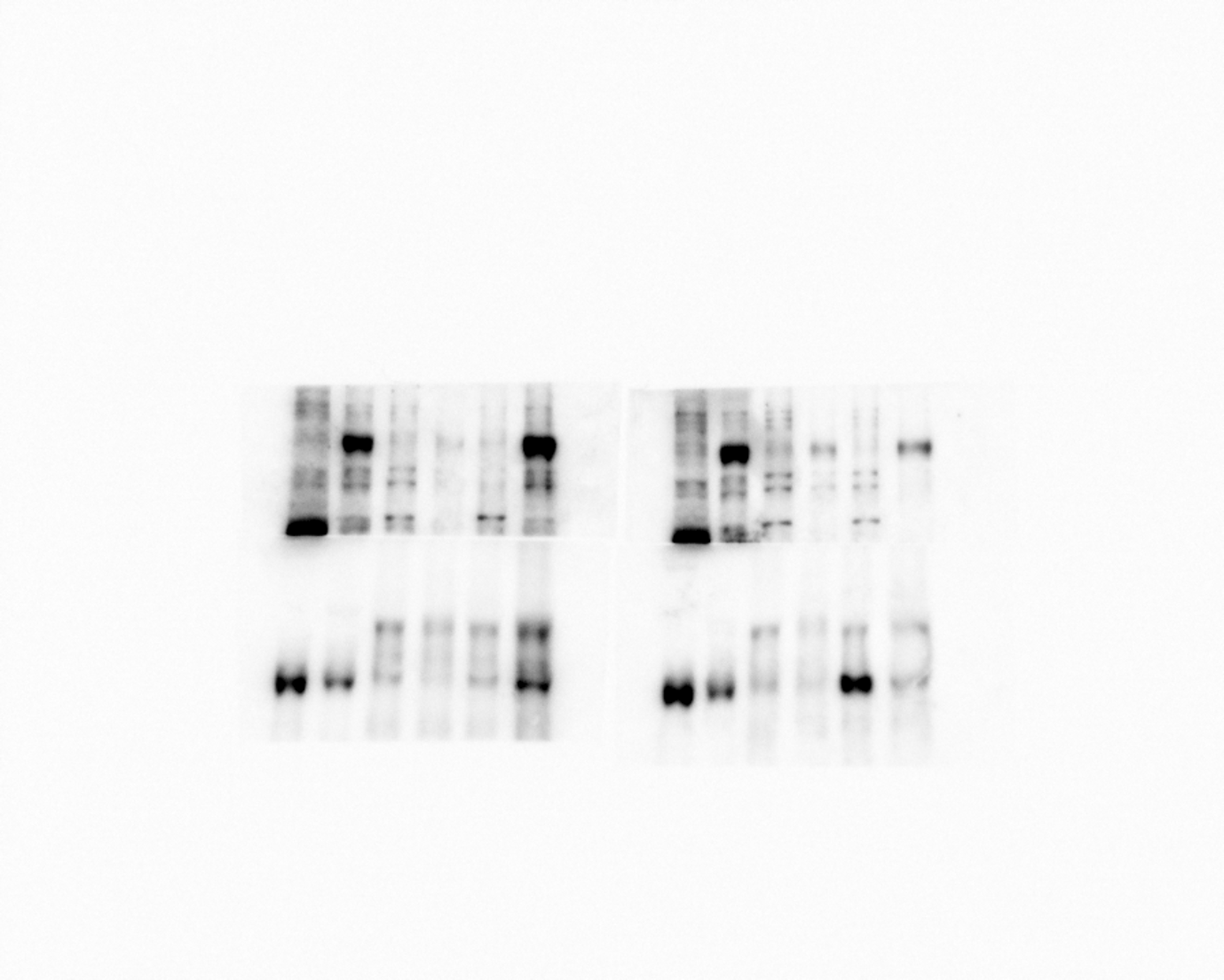

Supplement: Figure 3—source data 2. [file elife-102792-fig3-data2.zip › Figure 3-source data 2/Figure 3J_Source data 2/Figure 3J_Source data_anti NL2.tif]

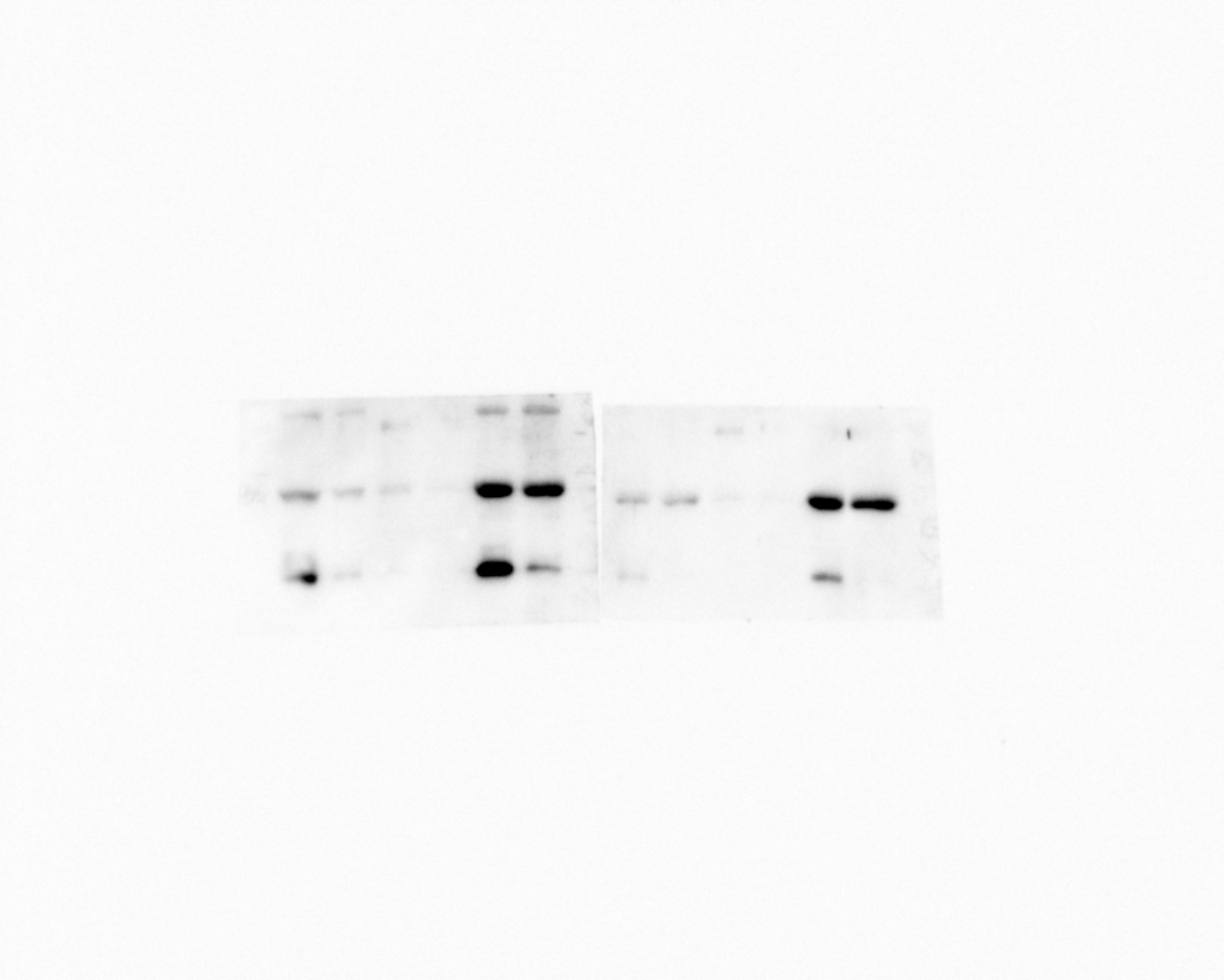

Supplement: Figure 3—source data 2. [file elife-102792-fig3-data2.zip › Figure 3-source data 2/Figure 3K_Source data 2/Figure 3K_Source data_Blot anti alpha1.tif]

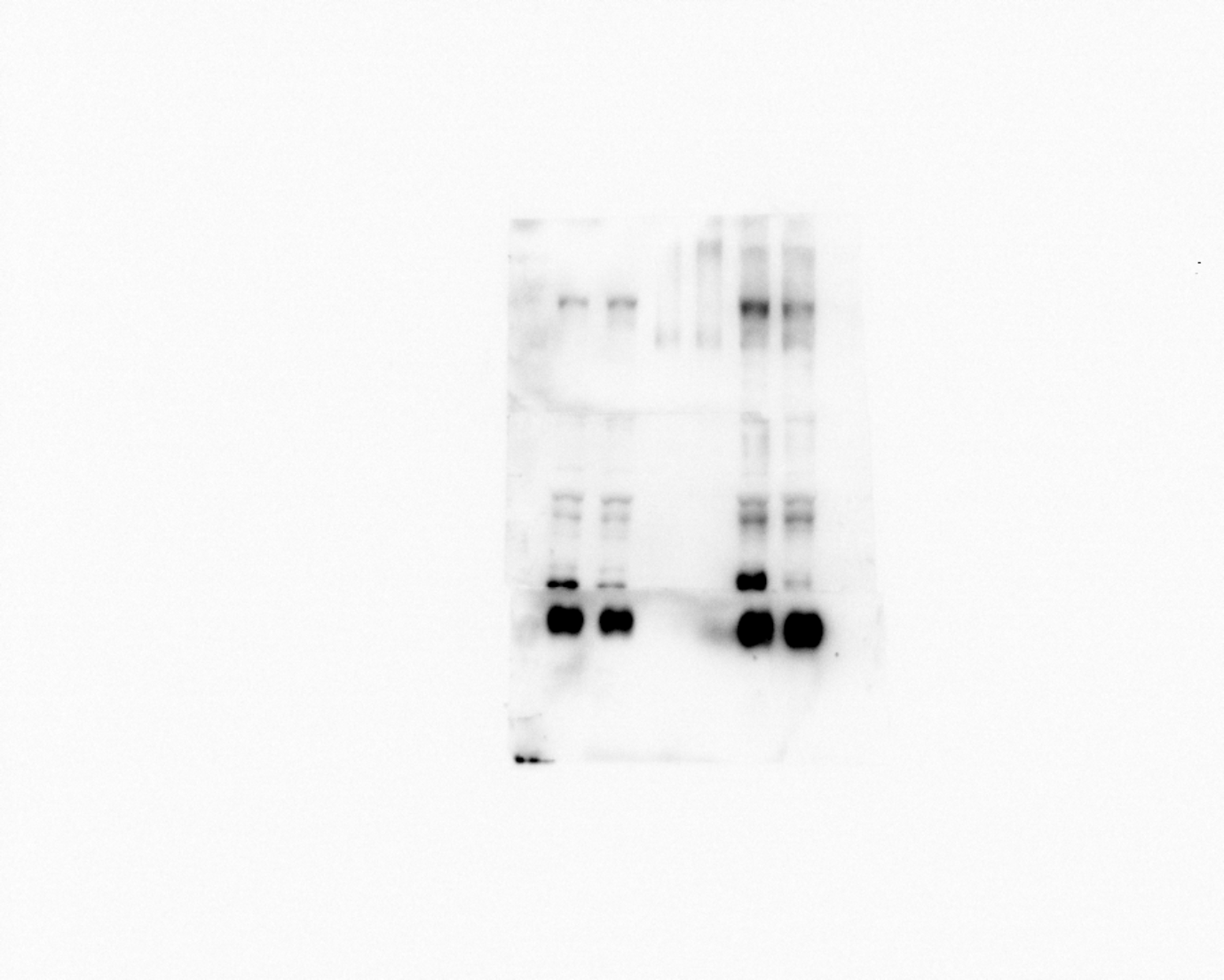

Supplement: Figure 3—source data 2. [file elife-102792-fig3-data2.zip › Figure 3-source data 2/Figure 3K_Source data 2/Figure 3K_Source data_Blot anti EndoA1.tif]

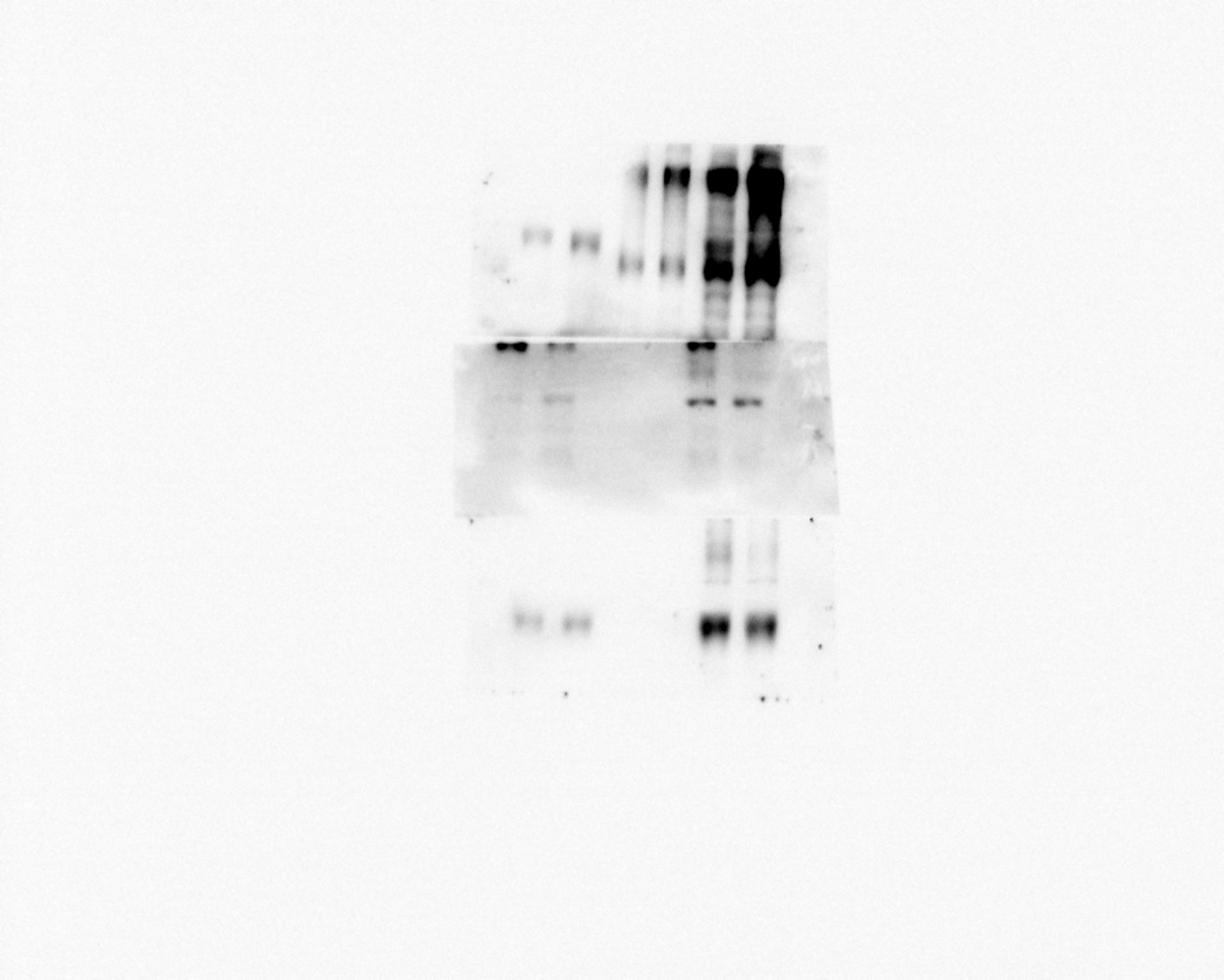

Supplement: Figure 3—source data 2. [file elife-102792-fig3-data2.zip › Figure 3-source data 2/Figure 3K_Source data 2/Figure 3K_Source data_Blot anti gamma2.tif]

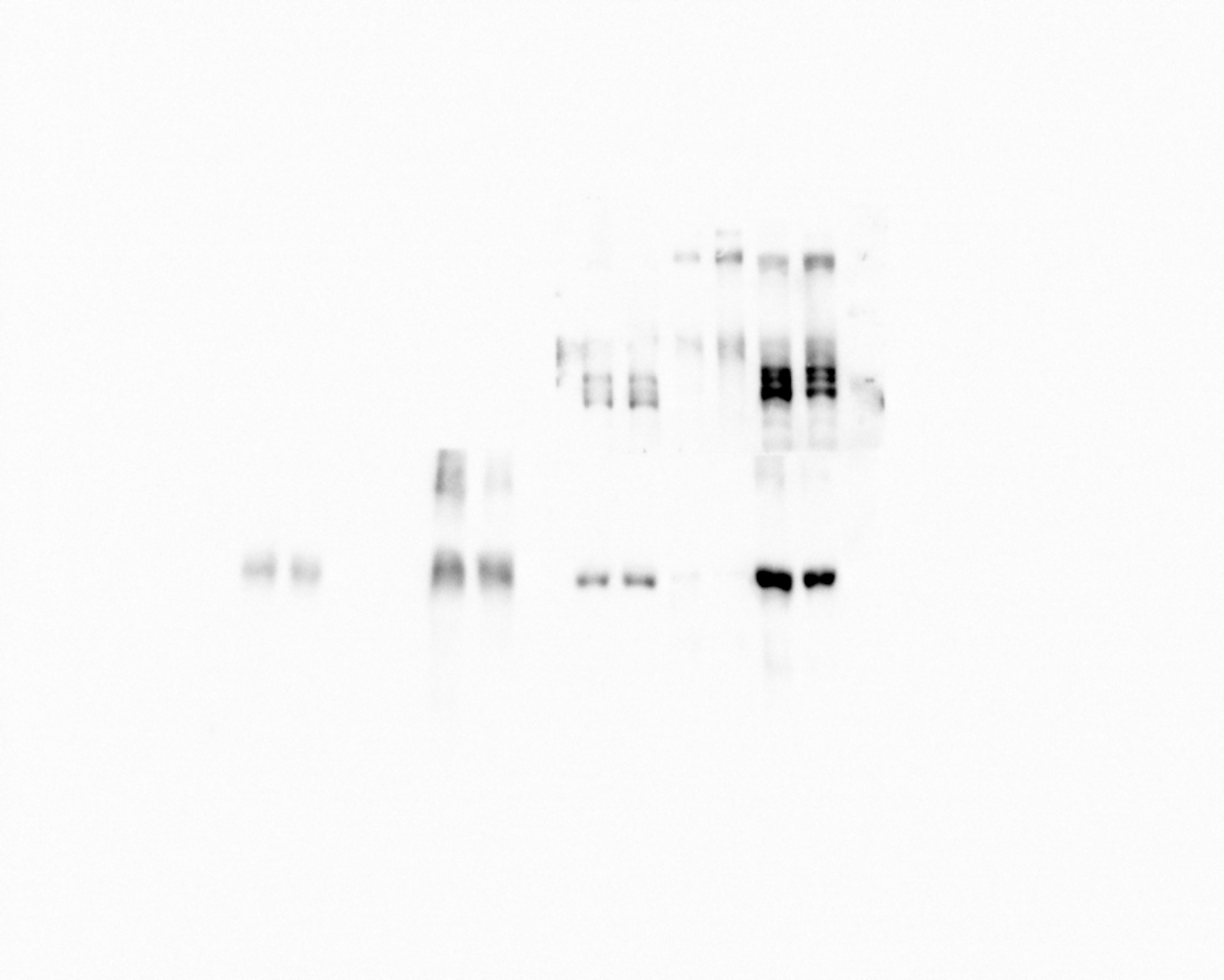

Supplement: Figure 3—source data 2. [file elife-102792-fig3-data2.zip › Figure 3-source data 2/Figure 3K_Source data 2/Figure 3K_Source data_Blot anti GPN.tif]

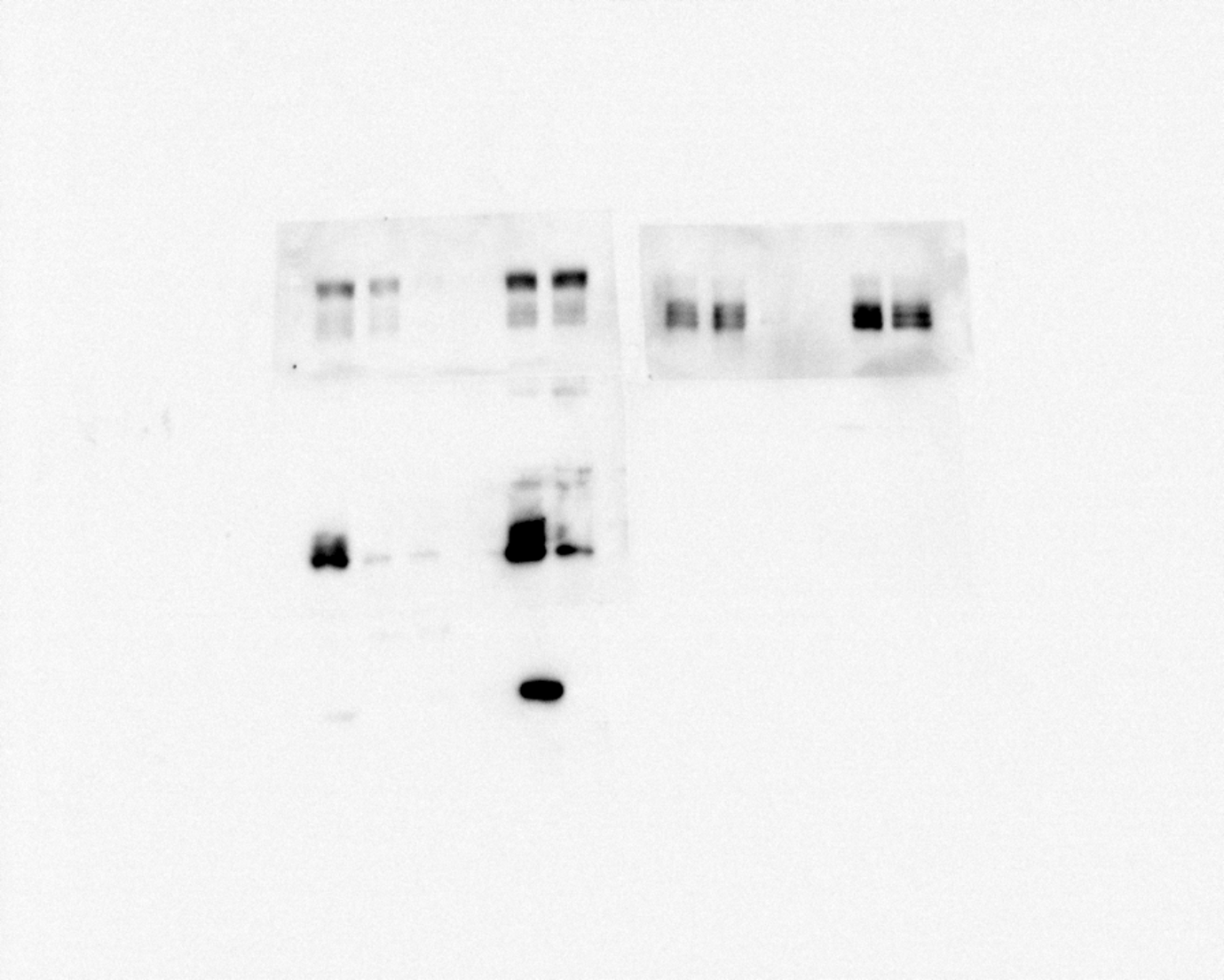

Supplement: Figure 3—source data 2. [file elife-102792-fig3-data2.zip › Figure 3-source data 2/Figure 3K_Source data 2/Figure 3K_Source data_Blot anti NLG2.tif]

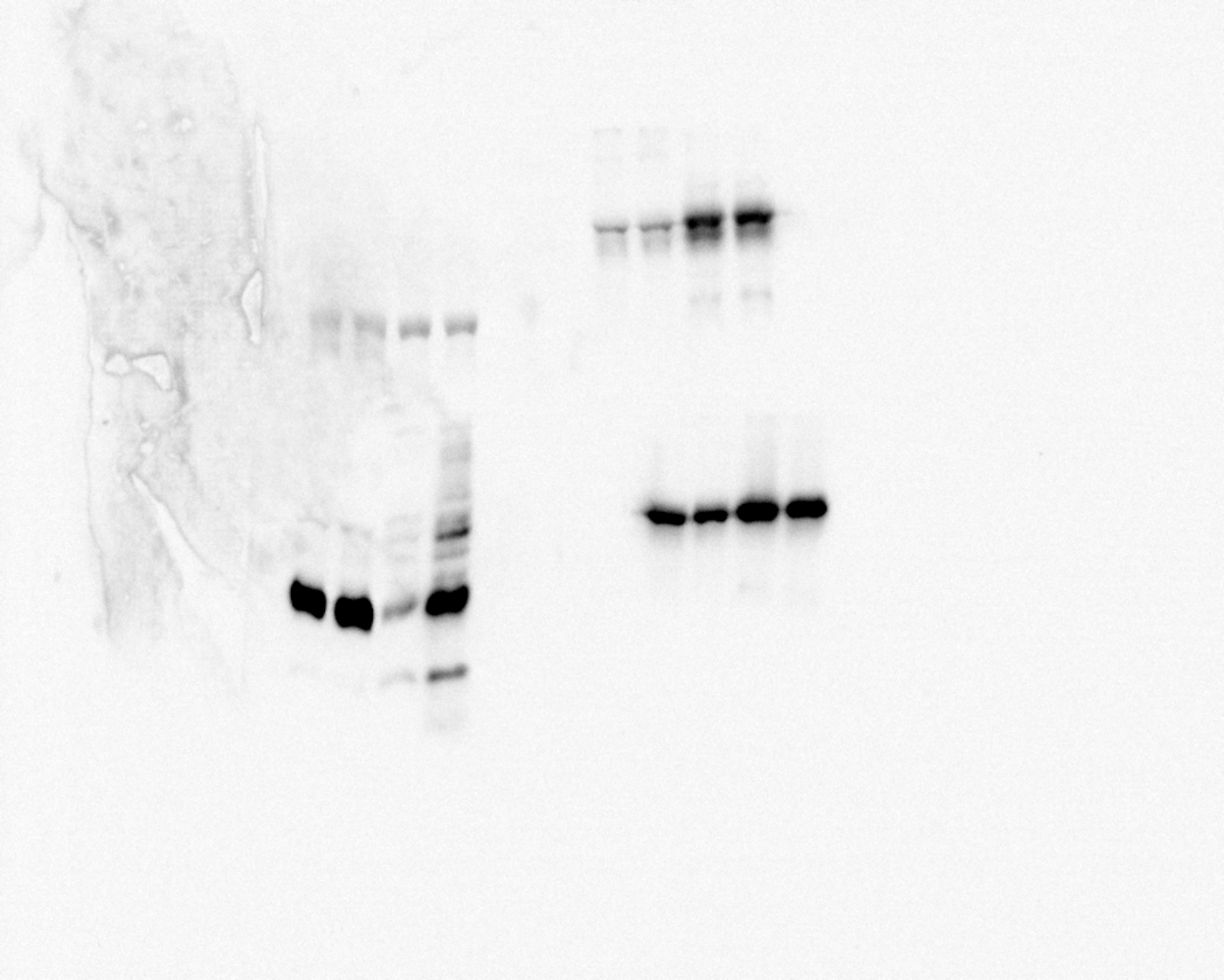

Supplement: Figure 3—source data 2. [file elife-102792-fig3-data2.zip › Figure 3-source data 2/Figure 3L_Source data2/Figure 3L_Source data_anti alpha1.tif]

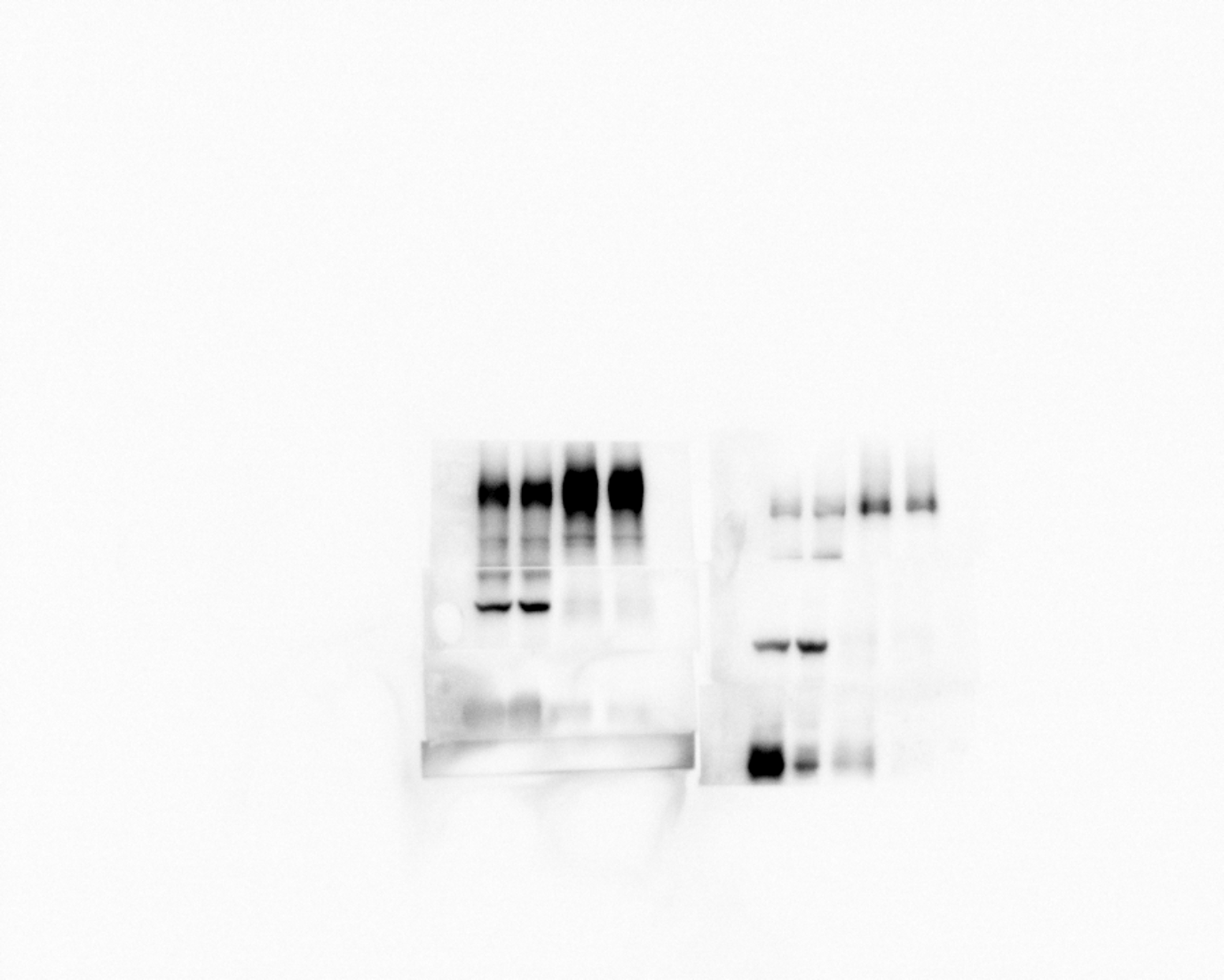

Supplement: Figure 3—source data 2. [file elife-102792-fig3-data2.zip › Figure 3-source data 2/Figure 3L_Source data2/Figure 3L_Source data_anti EndoA1.tif]

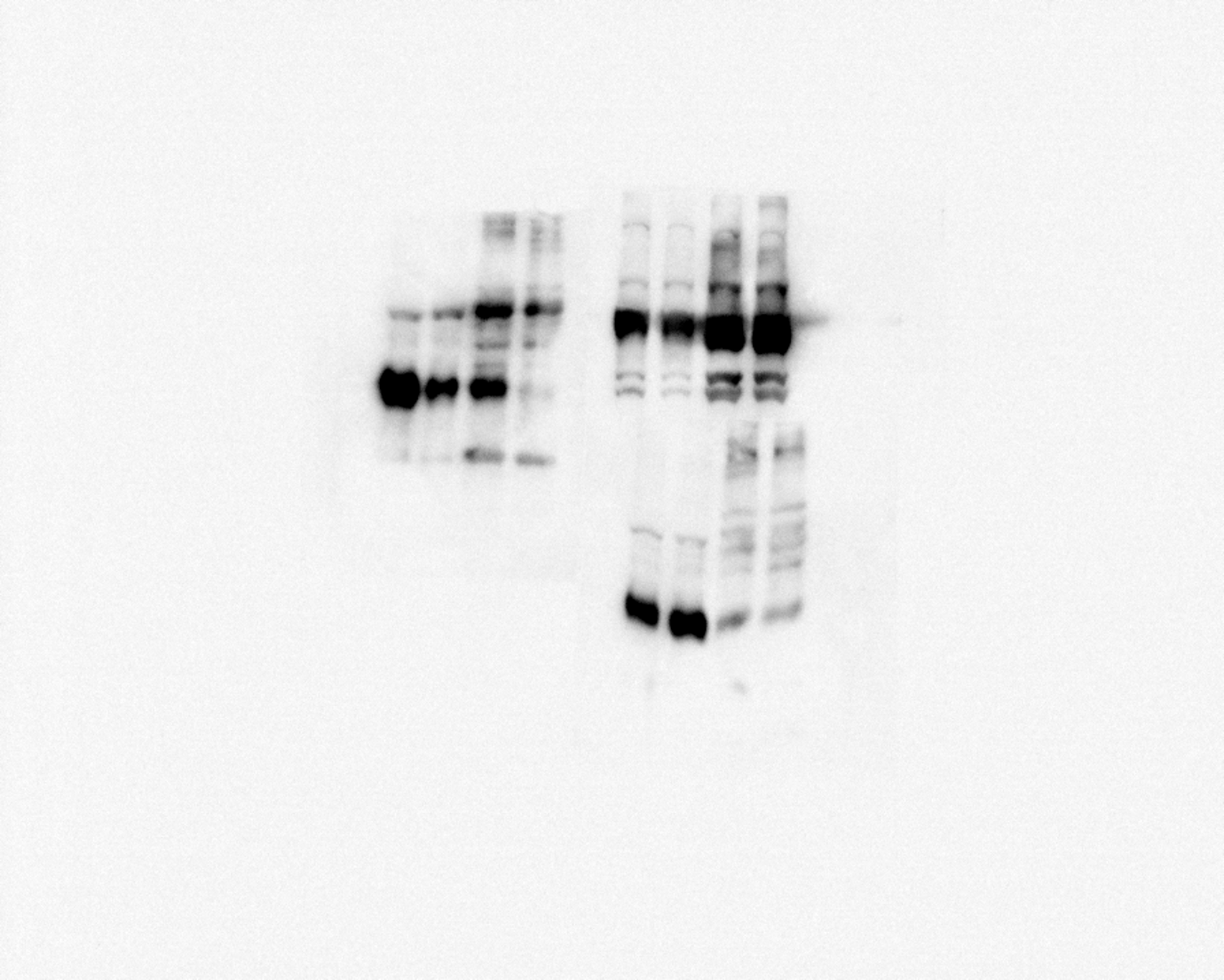

Supplement: Figure 3—source data 2. [file elife-102792-fig3-data2.zip › Figure 3-source data 2/Figure 3L_Source data2/Figure 3L_Source data_anti gamma2 and SYP.tif]

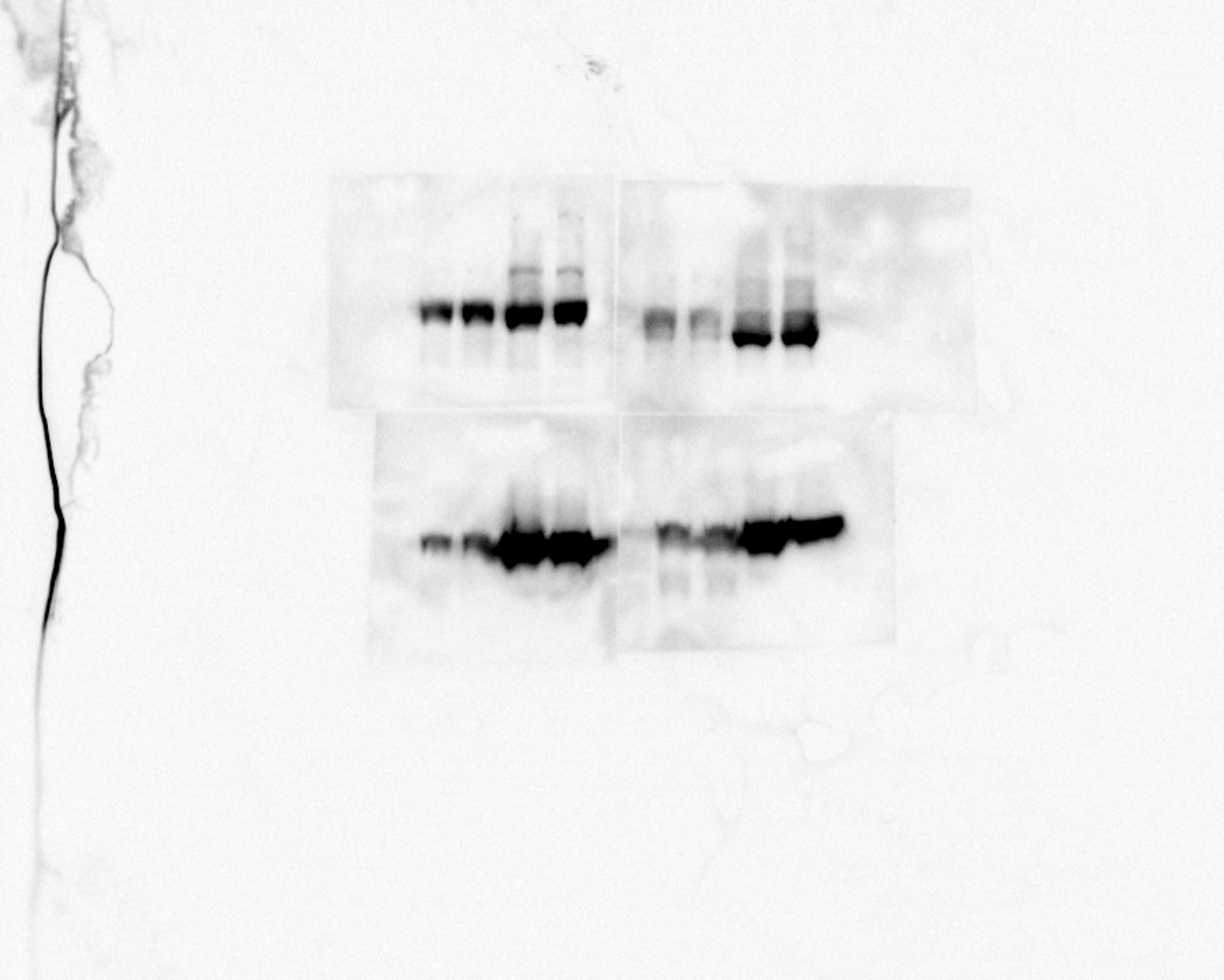

Supplement: Figure 3—source data 2. [file elife-102792-fig3-data2.zip › Figure 3-source data 2/Figure 3L_Source data2/Figure 3L_Source data_anti GluA1.tif]

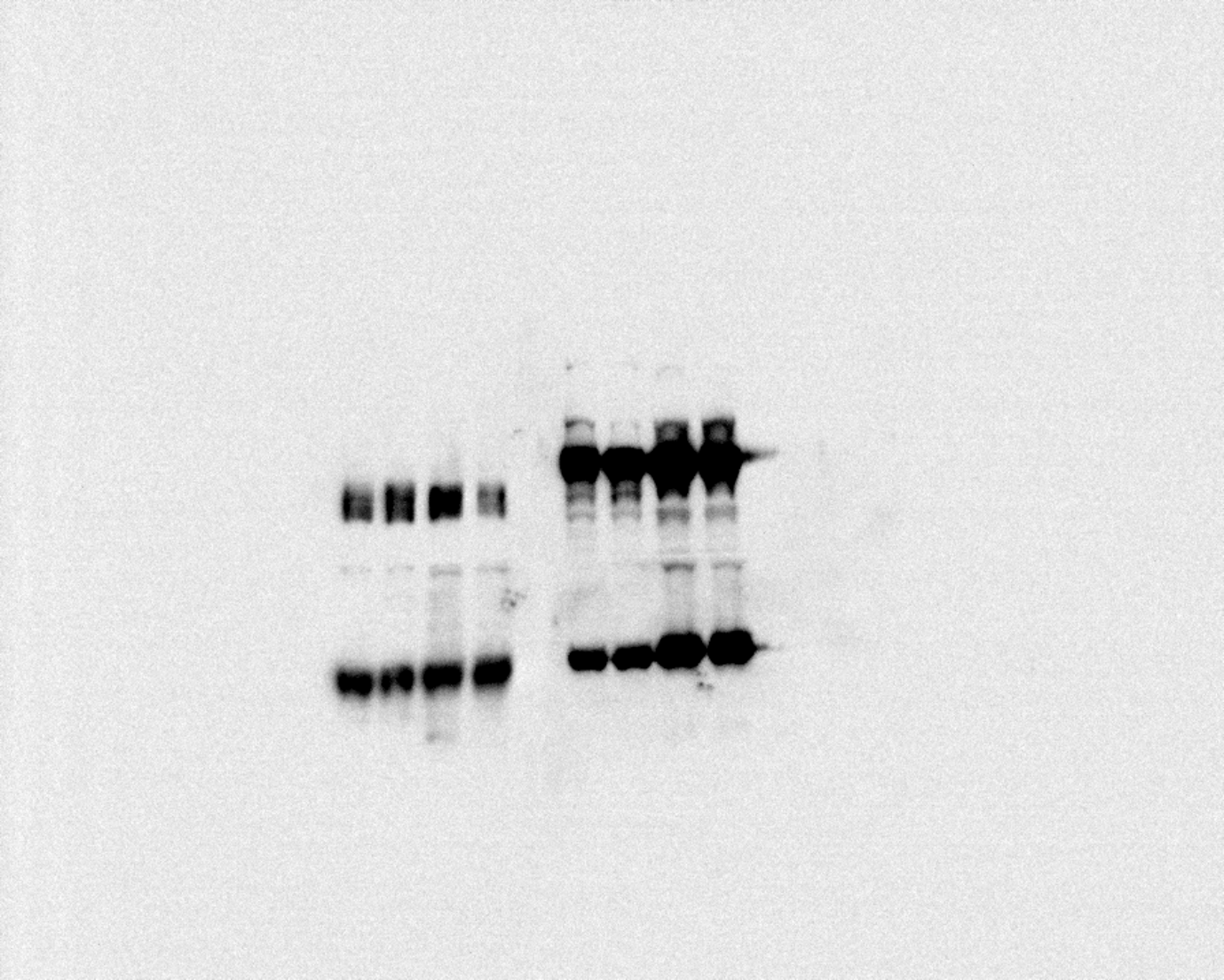

Supplement: Figure 3—source data 2. [file elife-102792-fig3-data2.zip › Figure 3-source data 2/Figure 3L_Source data2/Figure 3L_Source data_anti GPN.tif]

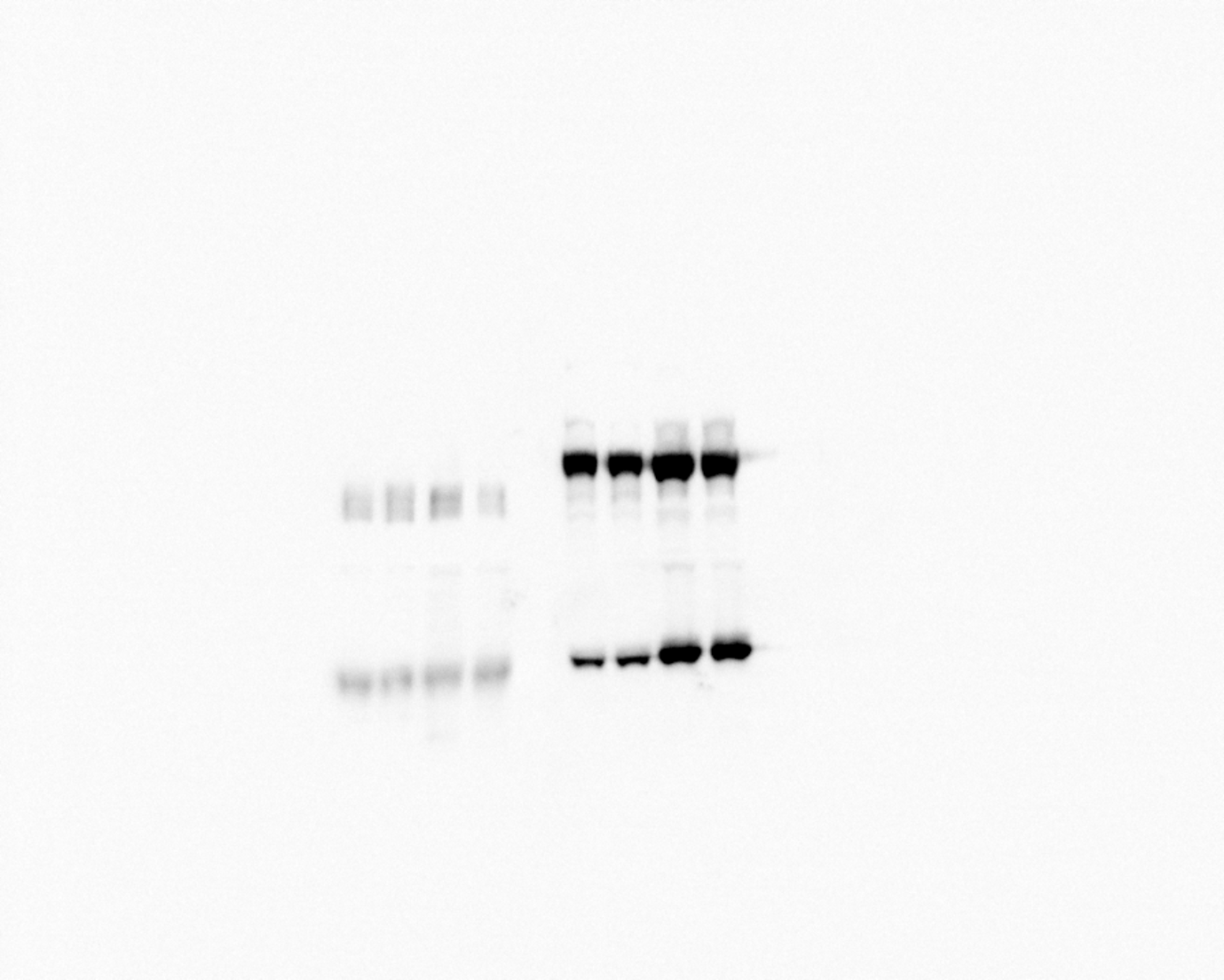

Supplement: Figure 3—source data 2. [file elife-102792-fig3-data2.zip › Figure 3-source data 2/Figure 3L_Source data2/Figure 3L_Source data_anti NL2.tif]

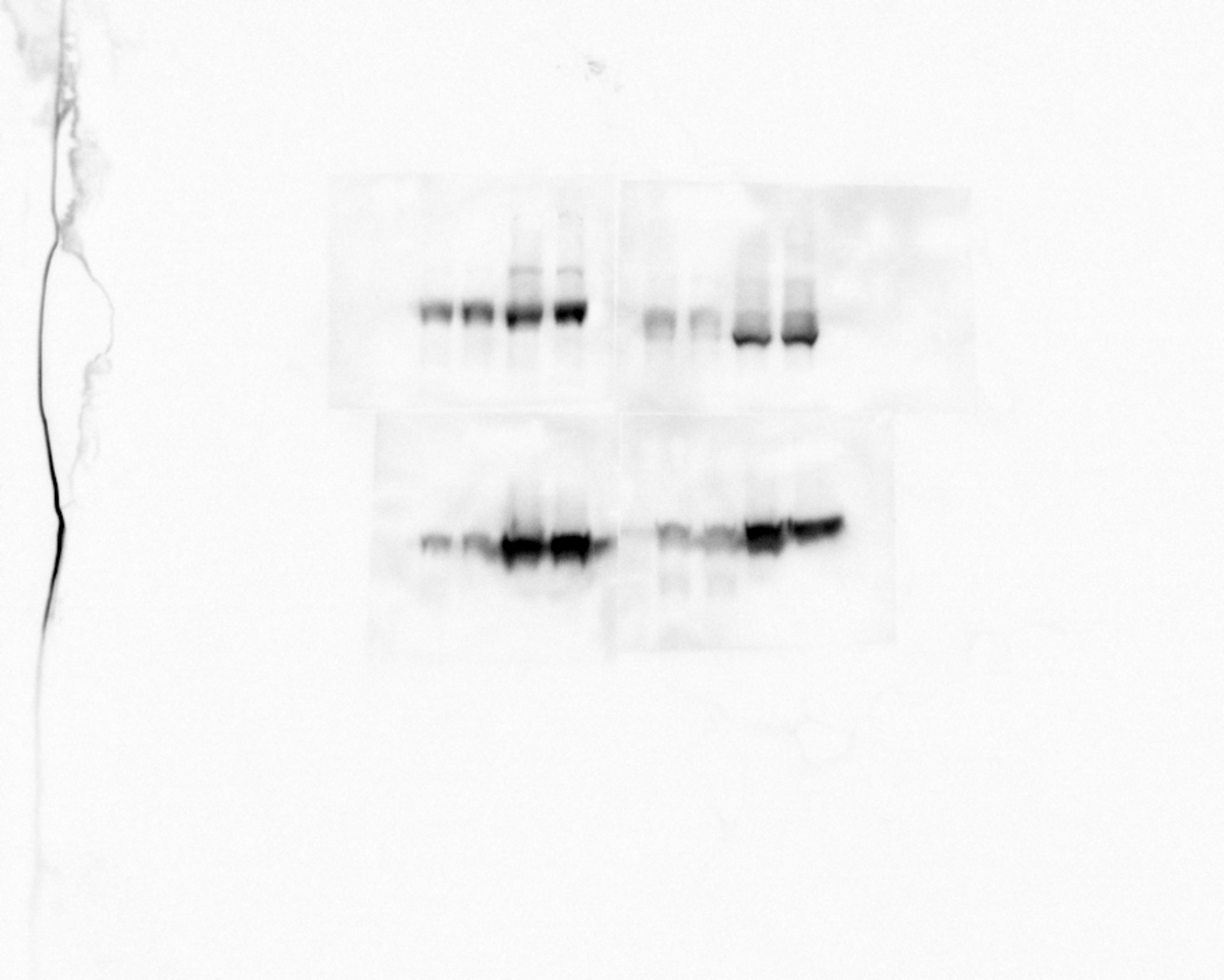

Supplement: Figure 3—source data 2. [file elife-102792-fig3-data2.zip › Figure 3-source data 2/Figure 3L_Source data2/Figure 3L_Source data_anti NR1.tif]

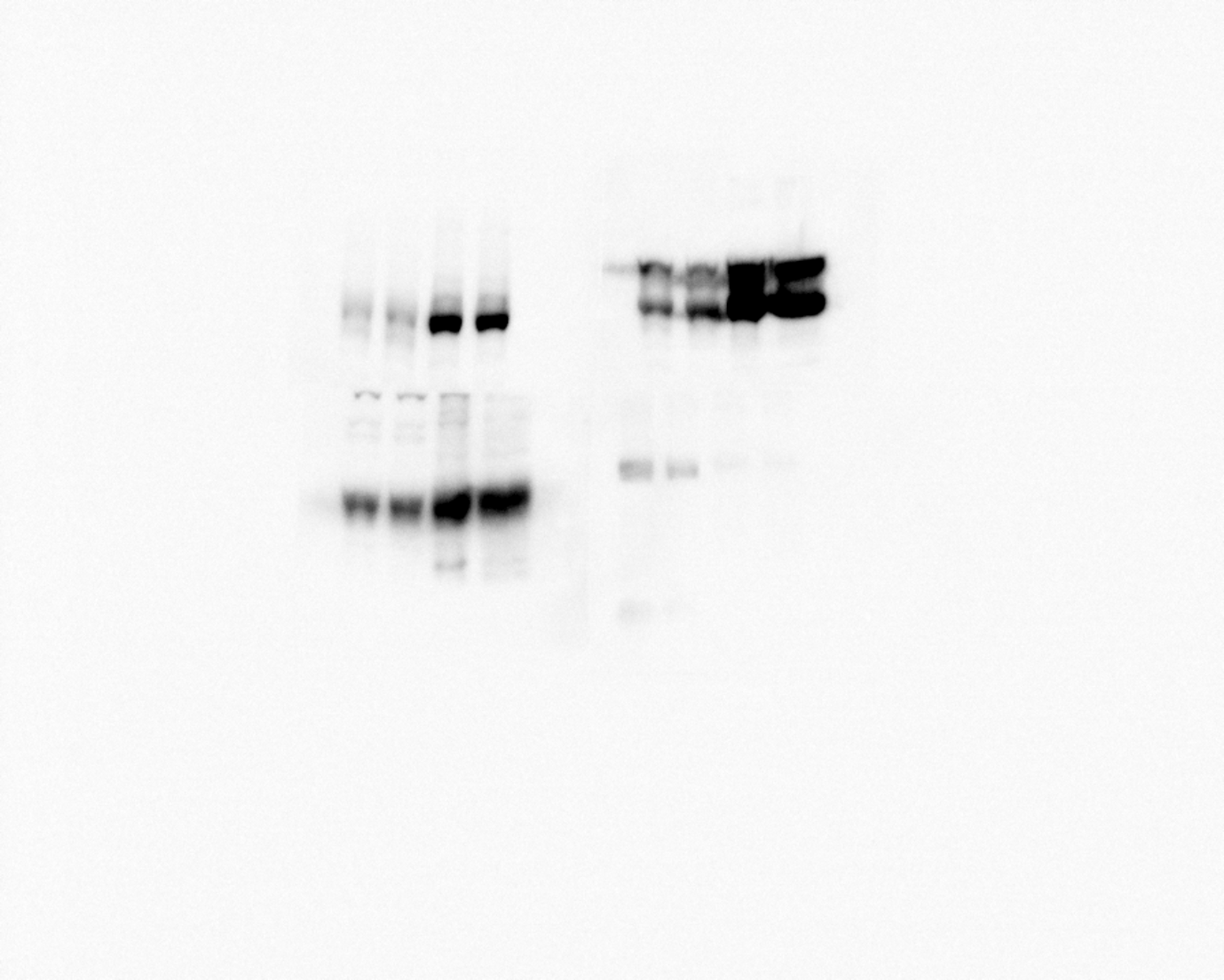

Supplement: Figure 3—source data 2. [file elife-102792-fig3-data2.zip › Figure 3-source data 2/Figure 3L_Source data2/Figure 3L_Source data_anti PSD95.tif]

Figure 4A, Source Data

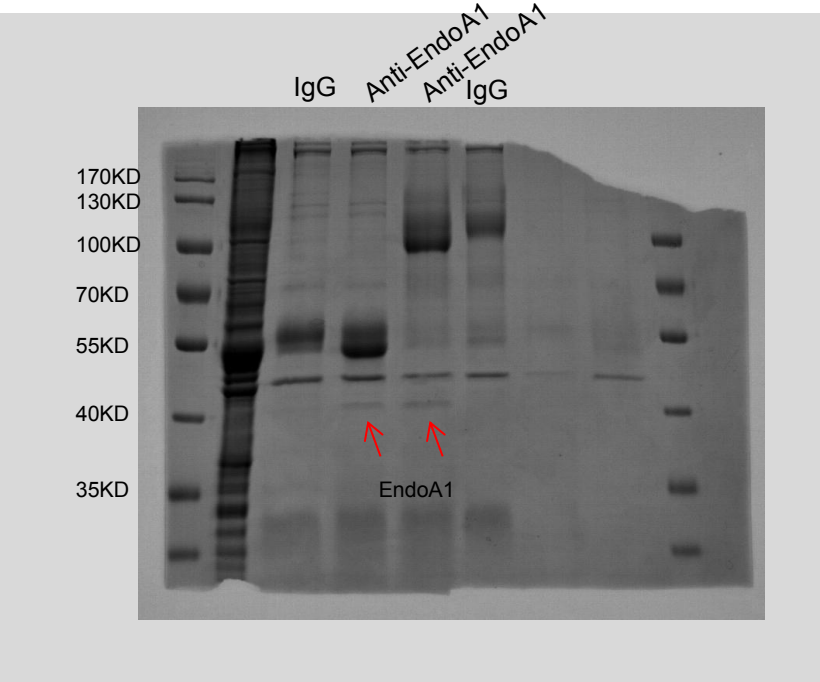

Supplement: Figure 4—source data 1. [file elife-102792-fig4-data1.zip › Figure 4-source data 1/Figure 4A-Source data 1.pdf]

Figure 4C, Source Data

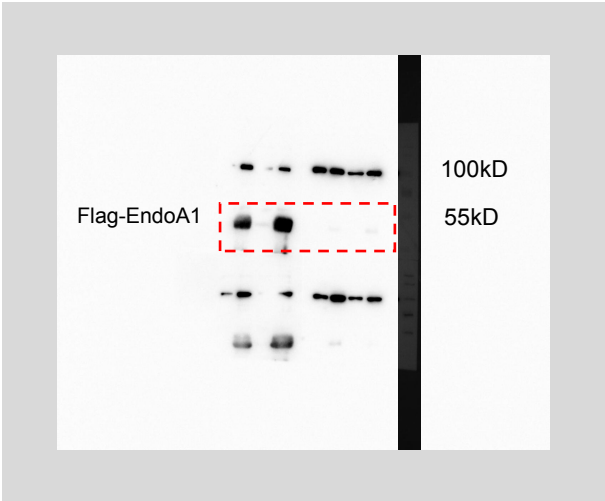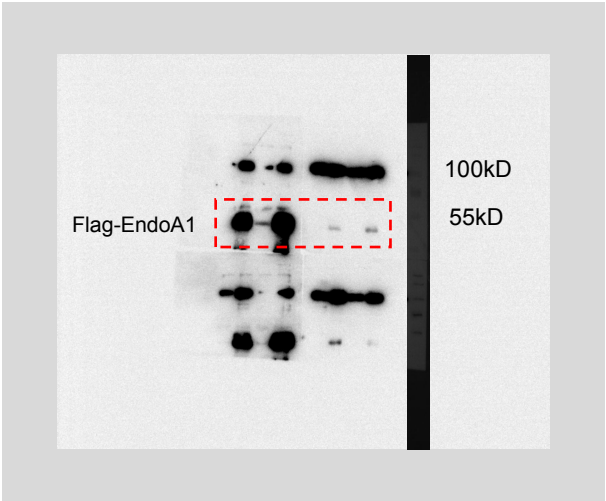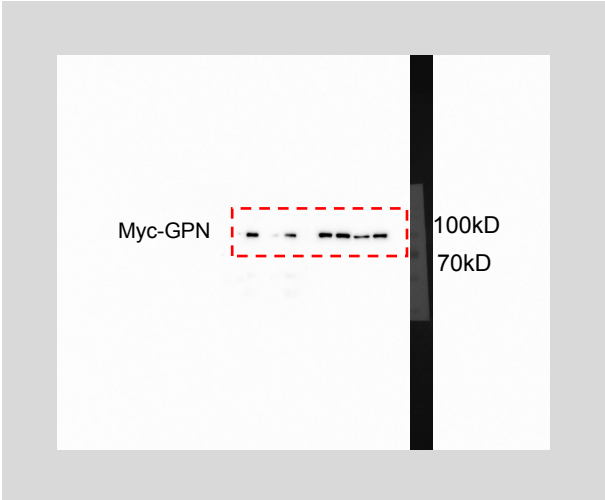

Supplement: Figure 4—source data 1. [file elife-102792-fig4-data1.zip › Figure 4-source data 1/Figure 4C_Source data 1.pdf]

Figure 4D, Source Data

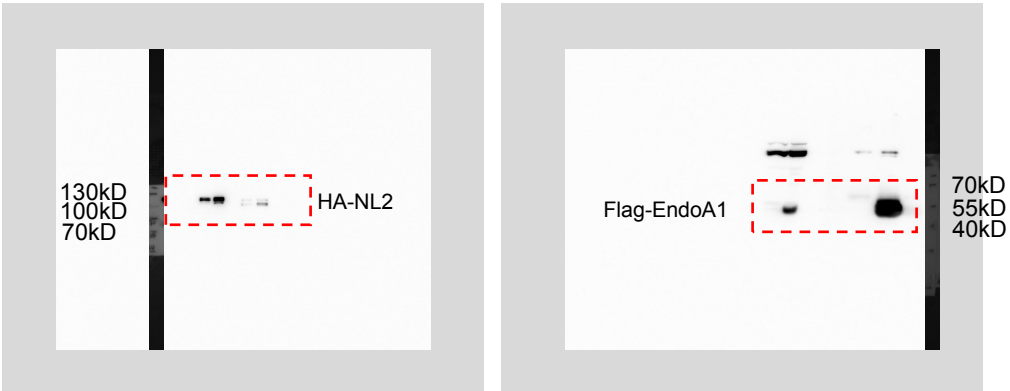

Supplement: Figure 4—source data 1. [file elife-102792-fig4-data1.zip › Figure 4-source data 1/Figure 4D_Source data 1.pdf]

Figure 4E, Source Data

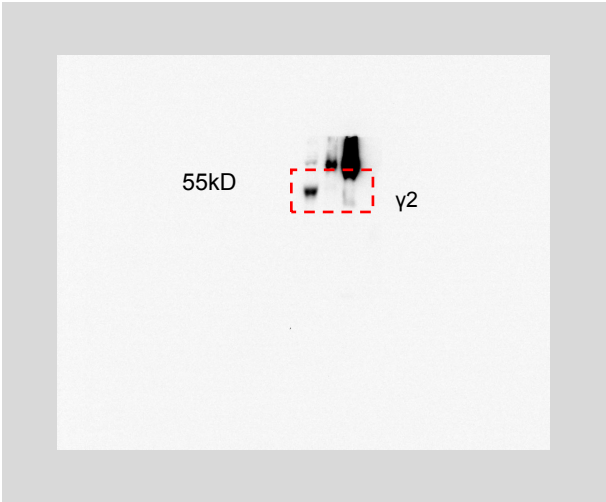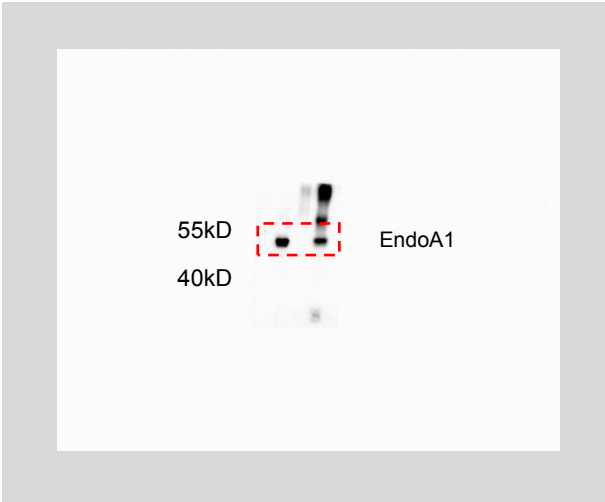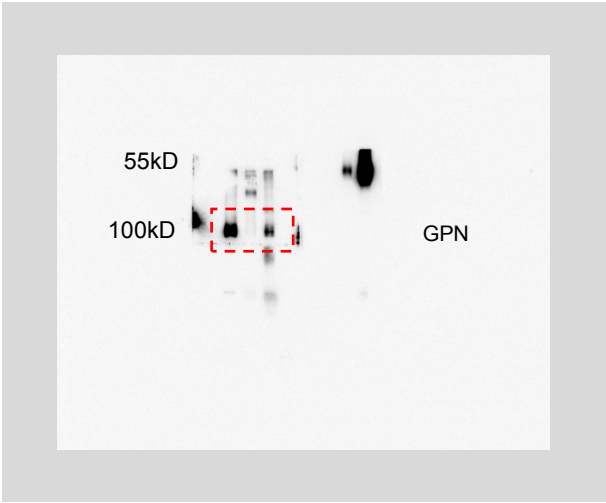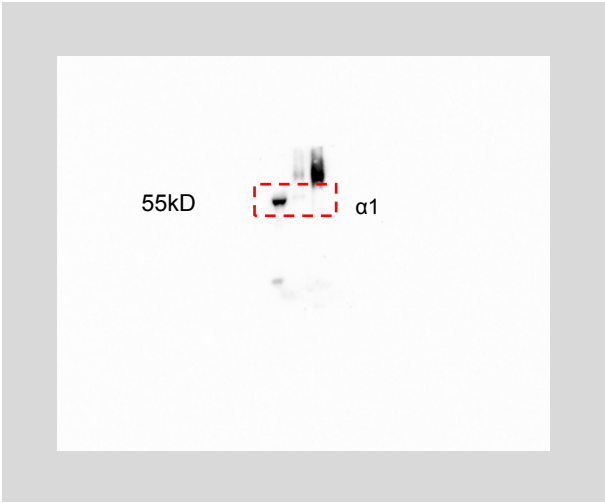

Supplement: Figure 4—source data 1. [file elife-102792-fig4-data1.zip › Figure 4-source data 1/Figure 4E_Source data 1.pdf]

Figure 4F, Source Data

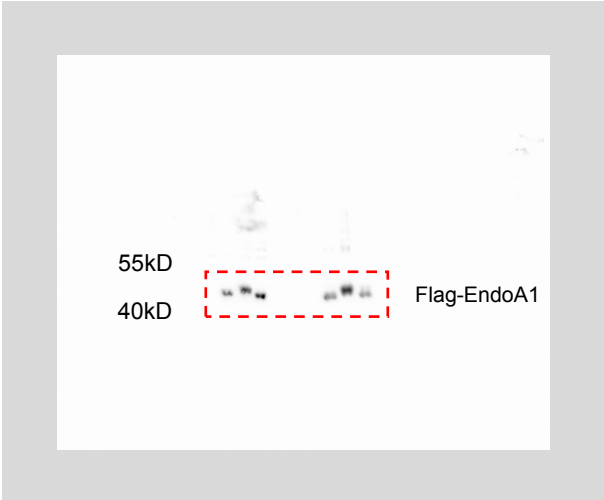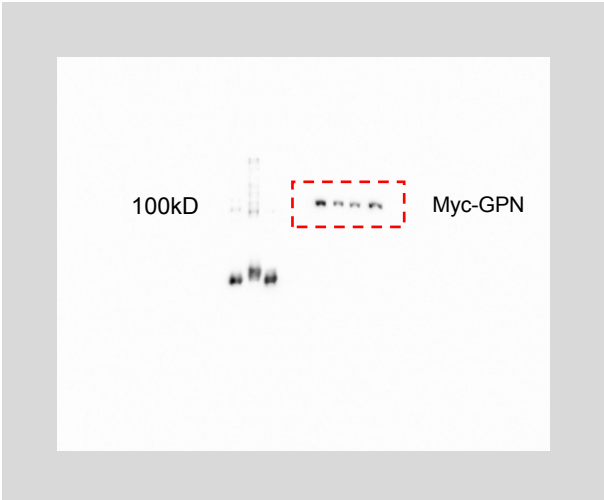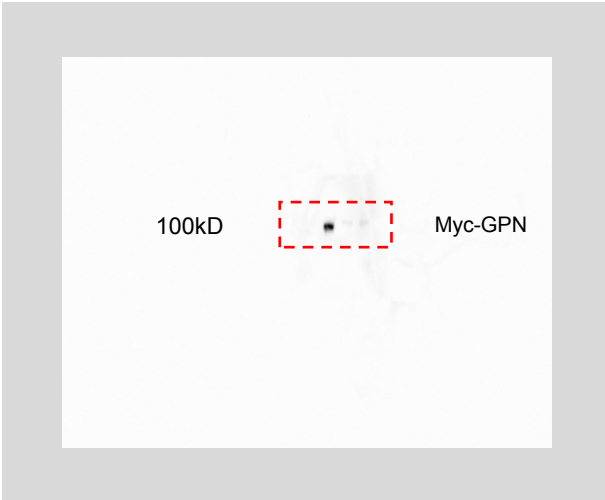

Supplement: Figure 4—source data 1. [file elife-102792-fig4-data1.zip › Figure 4-source data 1/Figure 4F_Source data 1.pdf]

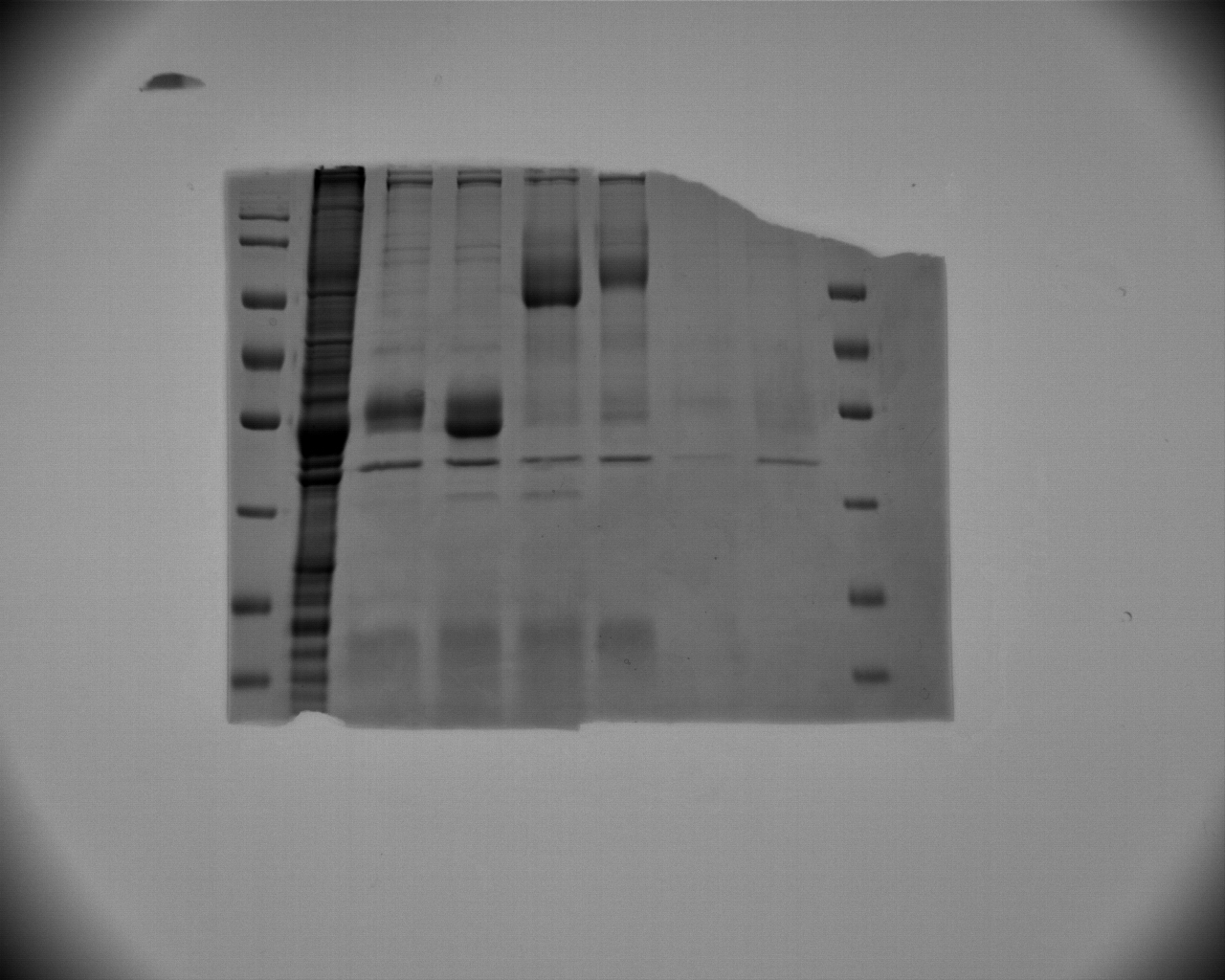

Supplement: Figure 4—source data 2. [file elife-102792-fig4-data2.zip › Figure 4-source data 2/Figure 4A_Source data 2/Figure 4A_Source data_Coomassie blue staining.tif]

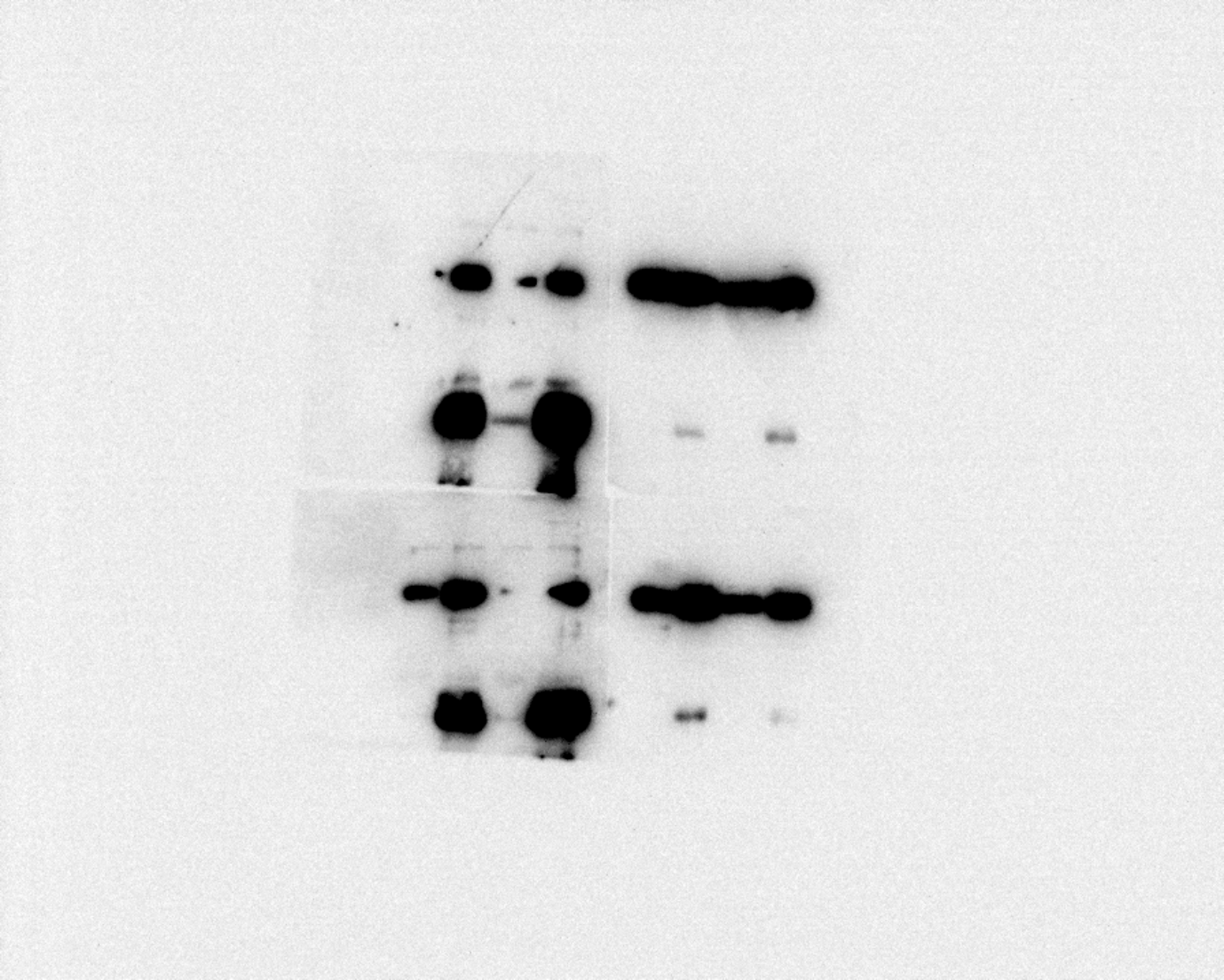

Supplement: Figure 4—source data 2. [file elife-102792-fig4-data2.zip › Figure 4-source data 2/Figure 4C_Source data 2/Figure 4C_Source data_blot anti Flag_EndoA1 LE.tif]

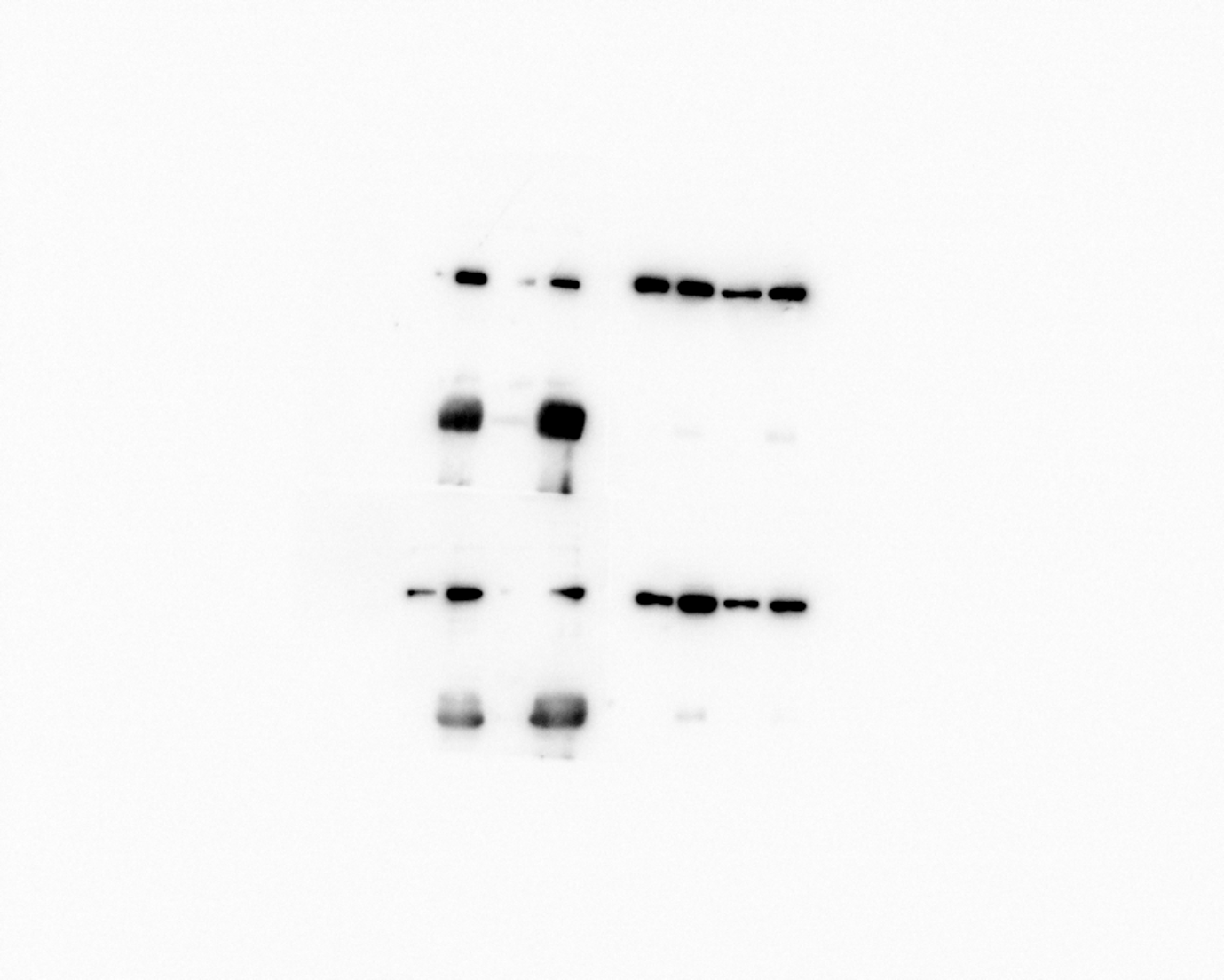

Supplement: Figure 4—source data 2. [file elife-102792-fig4-data2.zip › Figure 4-source data 2/Figure 4C_Source data 2/Figure 4C_Source data_blot anti Flag_EndoA1.tif]

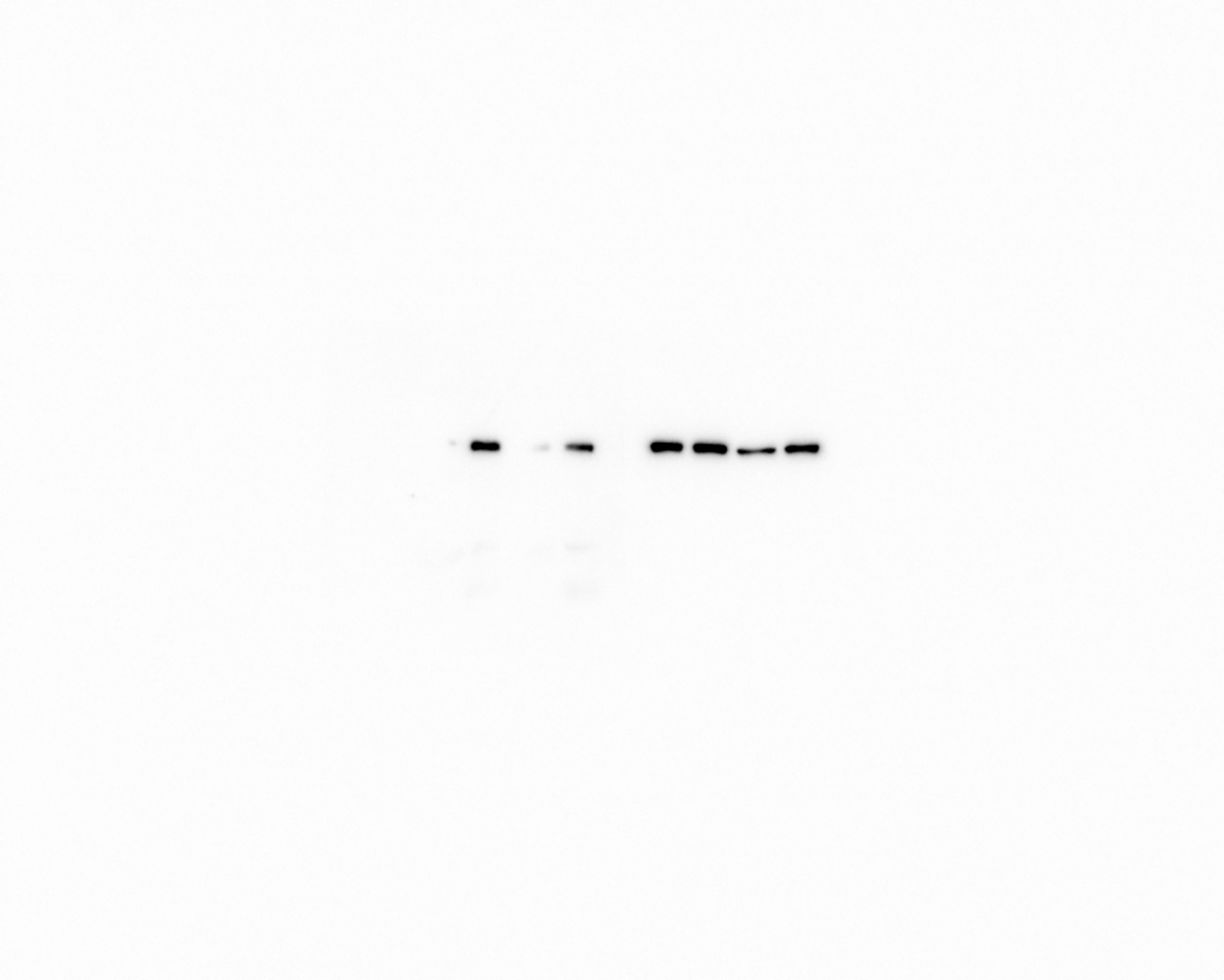

Supplement: Figure 4—source data 2. [file elife-102792-fig4-data2.zip › Figure 4-source data 2/Figure 4C_Source data 2/Figure 4C_Source data_blot anti myc_GPN 1.tif]

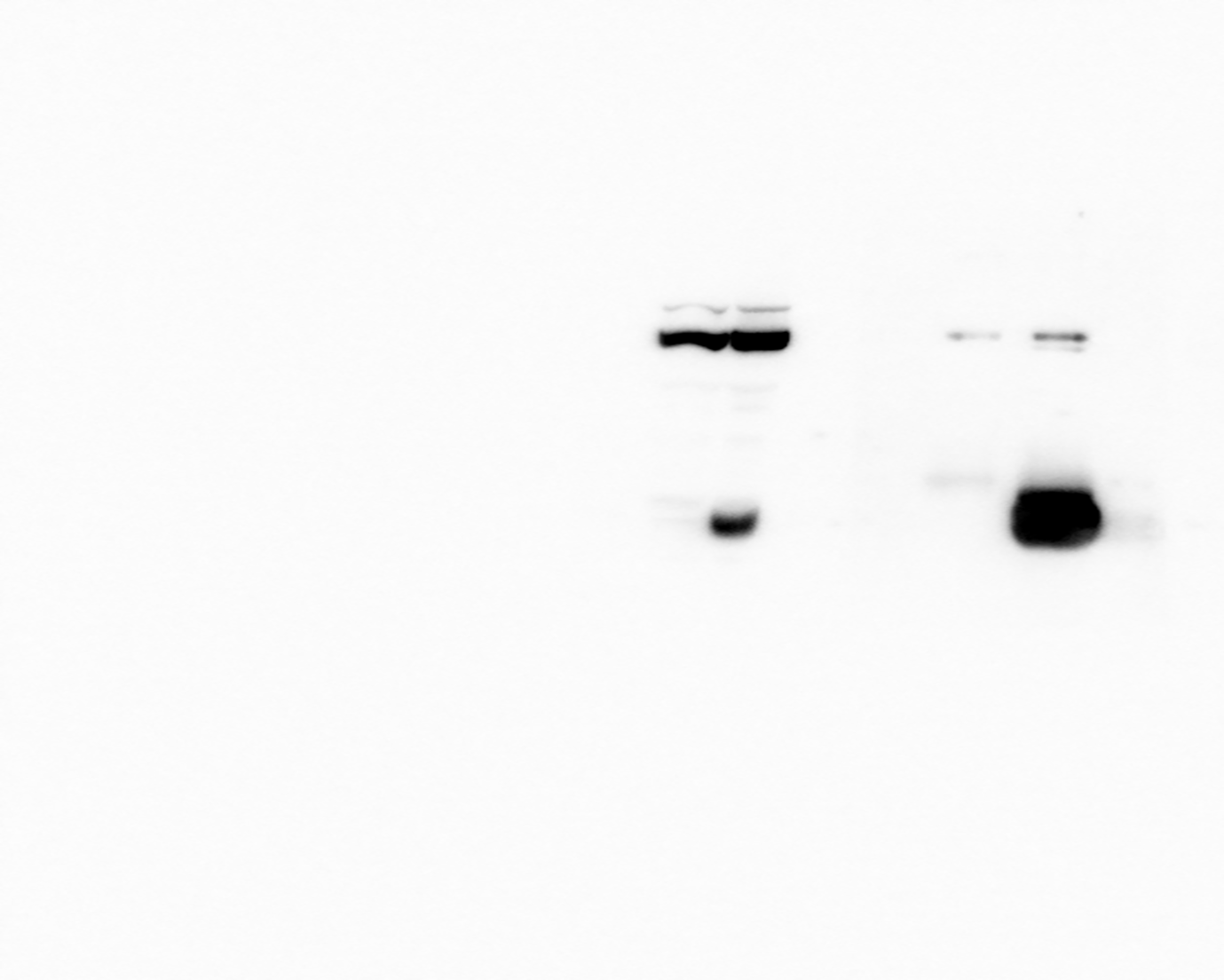

Supplement: Figure 4—source data 2. [file elife-102792-fig4-data2.zip › Figure 4-source data 2/Figure 4D_Source data 2/Figure 4D_Source data_blot anti Flag-EndoA1.tif]

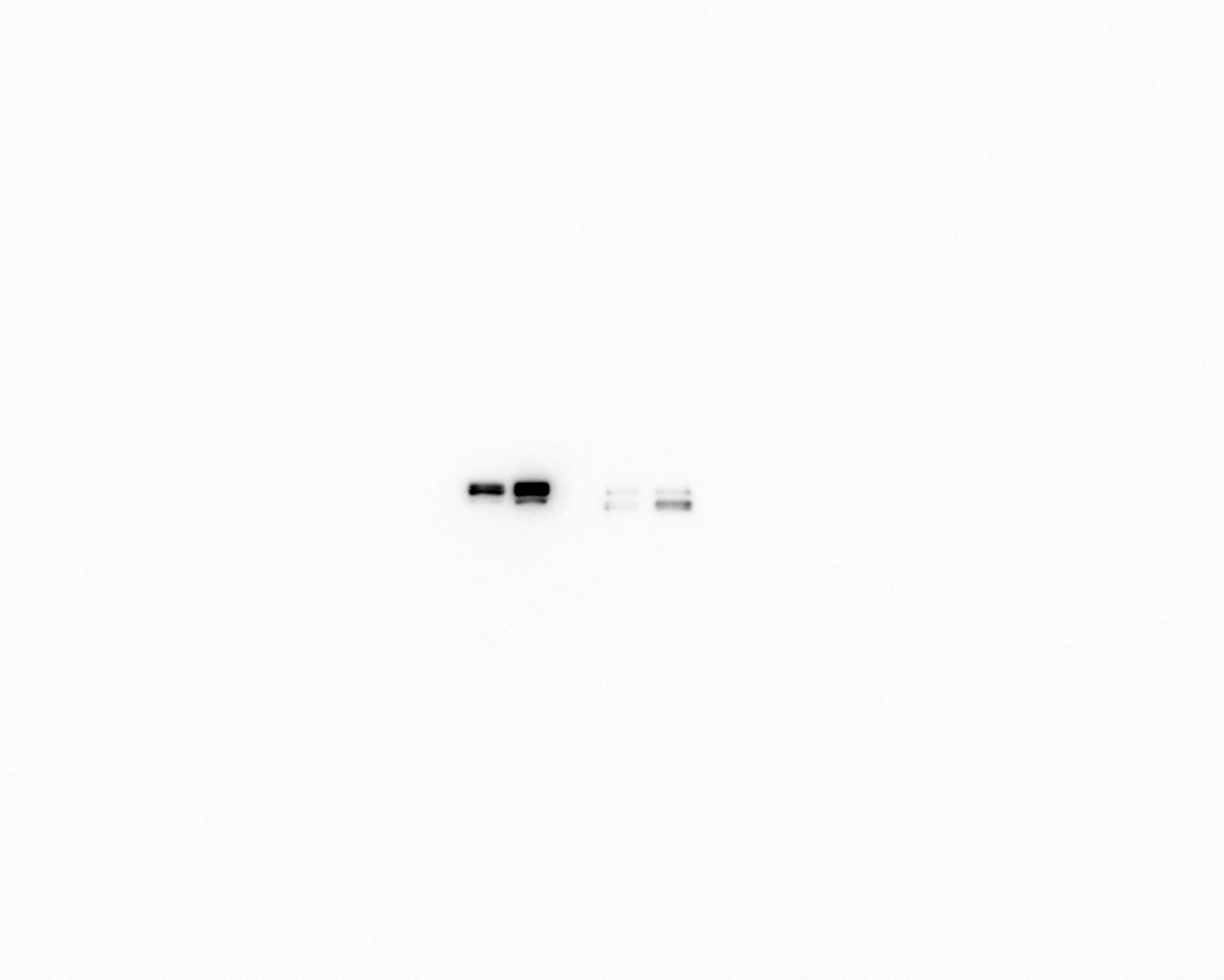

Supplement: Figure 4—source data 2. [file elife-102792-fig4-data2.zip › Figure 4-source data 2/Figure 4D_Source data 2/Figure 4D_Source data_blot anti HA-NL2.tif]

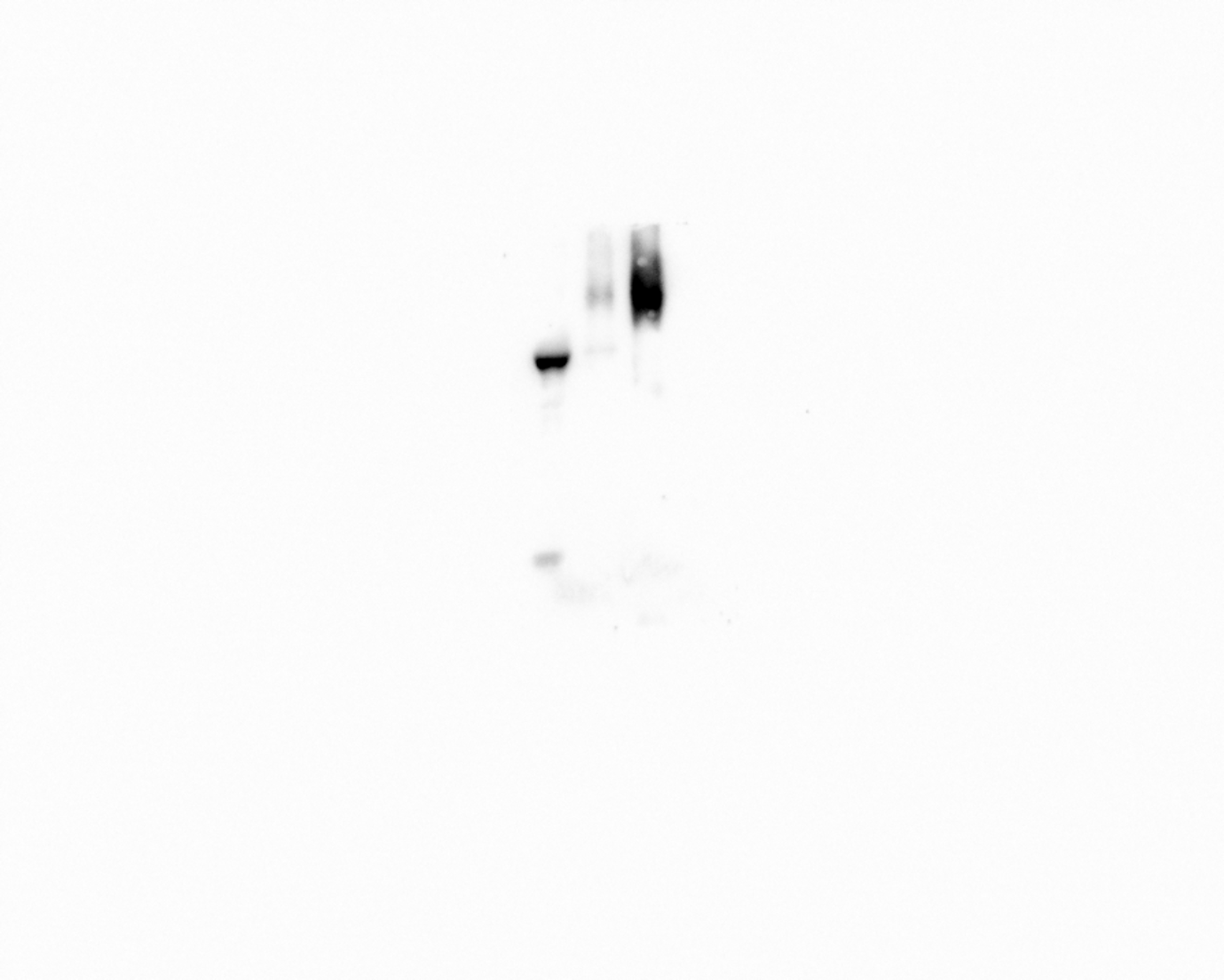

Supplement: Figure 4—source data 2. [file elife-102792-fig4-data2.zip › Figure 4-source data 2/Figure 4E_Source data 2/Figure 4E_Source data_anti alpha1.tif]

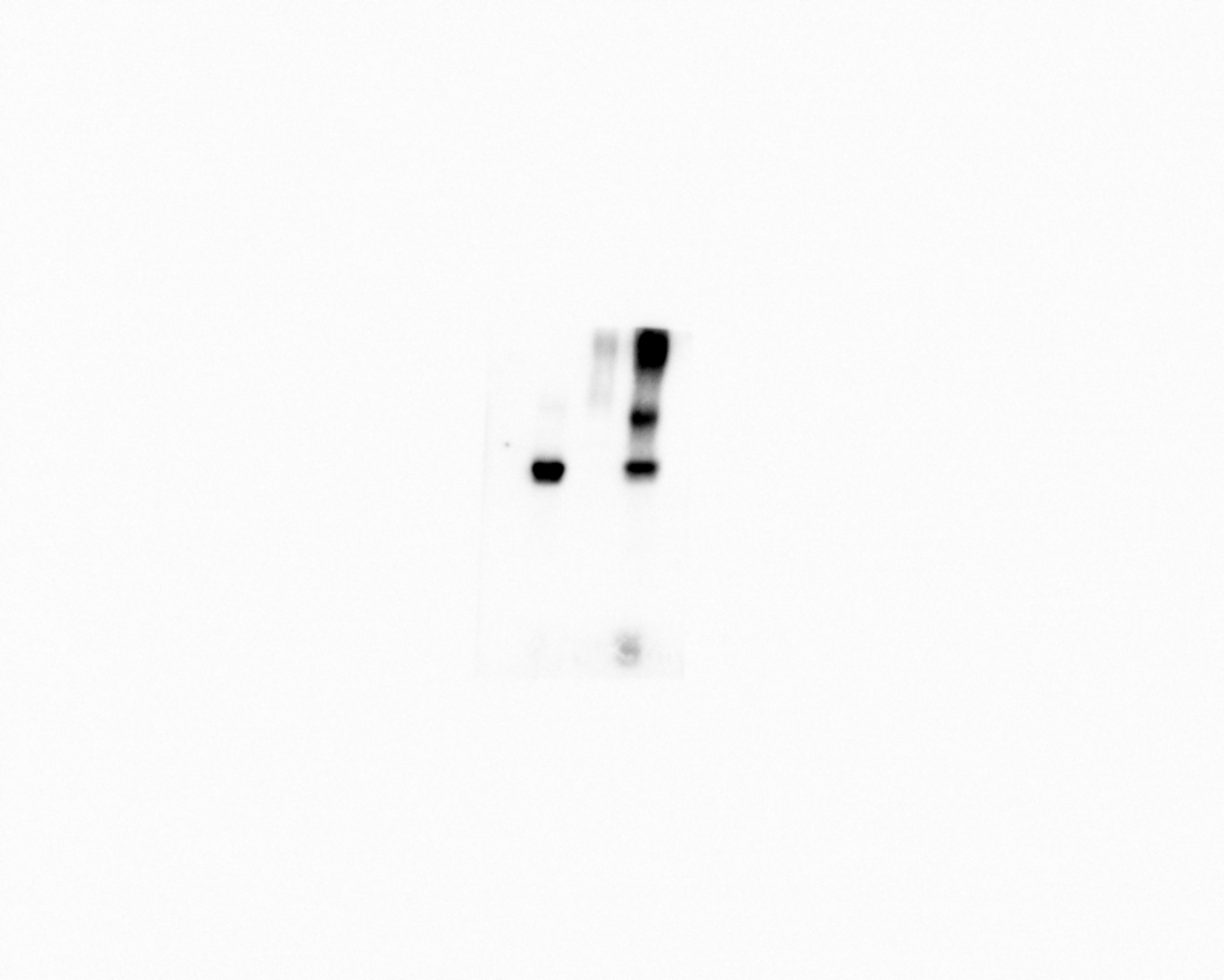

Supplement: Figure 4—source data 2. [file elife-102792-fig4-data2.zip › Figure 4-source data 2/Figure 4E_Source data 2/Figure 4E_Source data_anti EndoA1.tif]

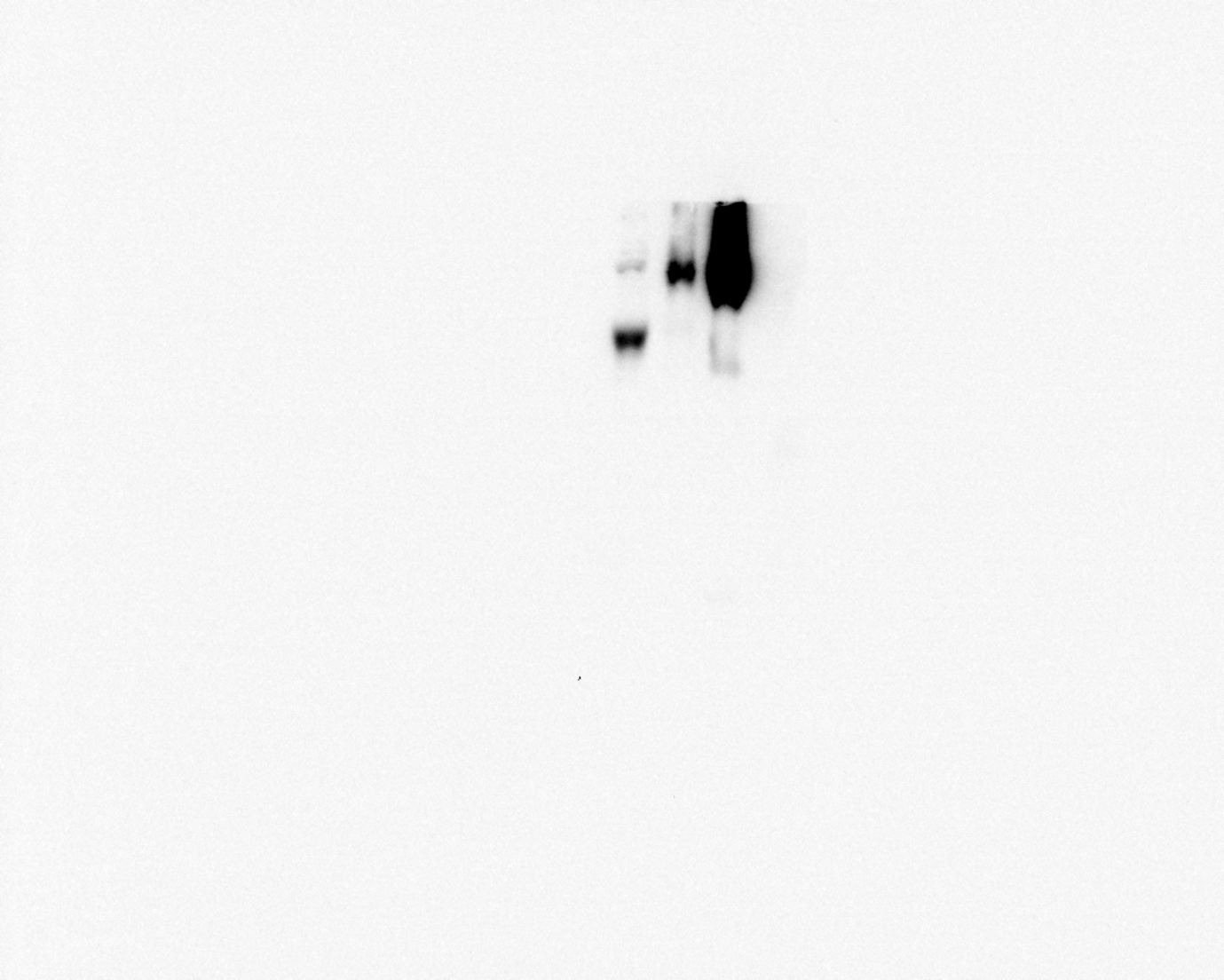

Supplement: Figure 4—source data 2. [file elife-102792-fig4-data2.zip › Figure 4-source data 2/Figure 4E_Source data 2/Figure 4E_Source data_anti gamma2.tif]

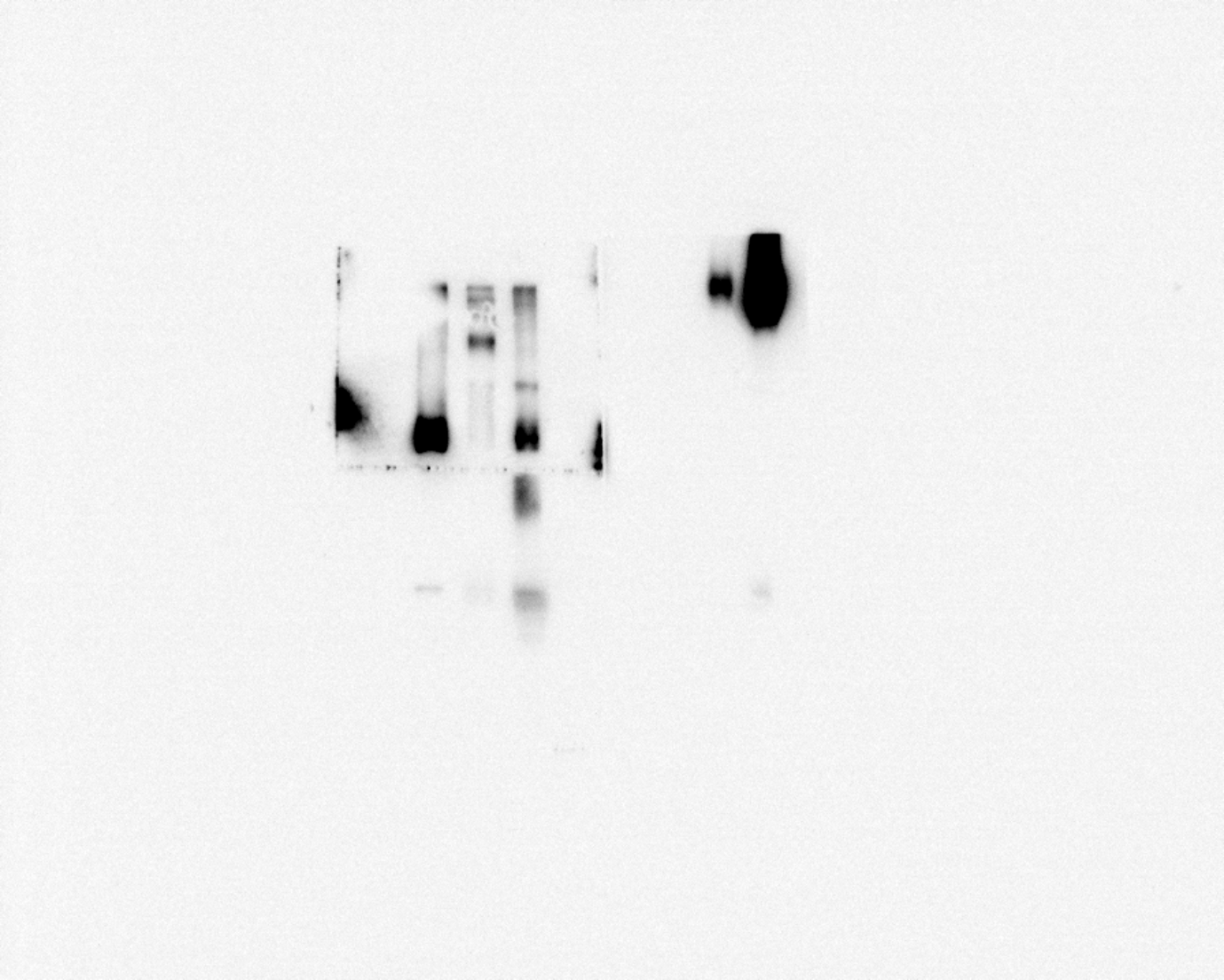

Supplement: Figure 4—source data 2. [file elife-102792-fig4-data2.zip › Figure 4-source data 2/Figure 4E_Source data 2/Figure 4E_Source data_anti GPN.tif]

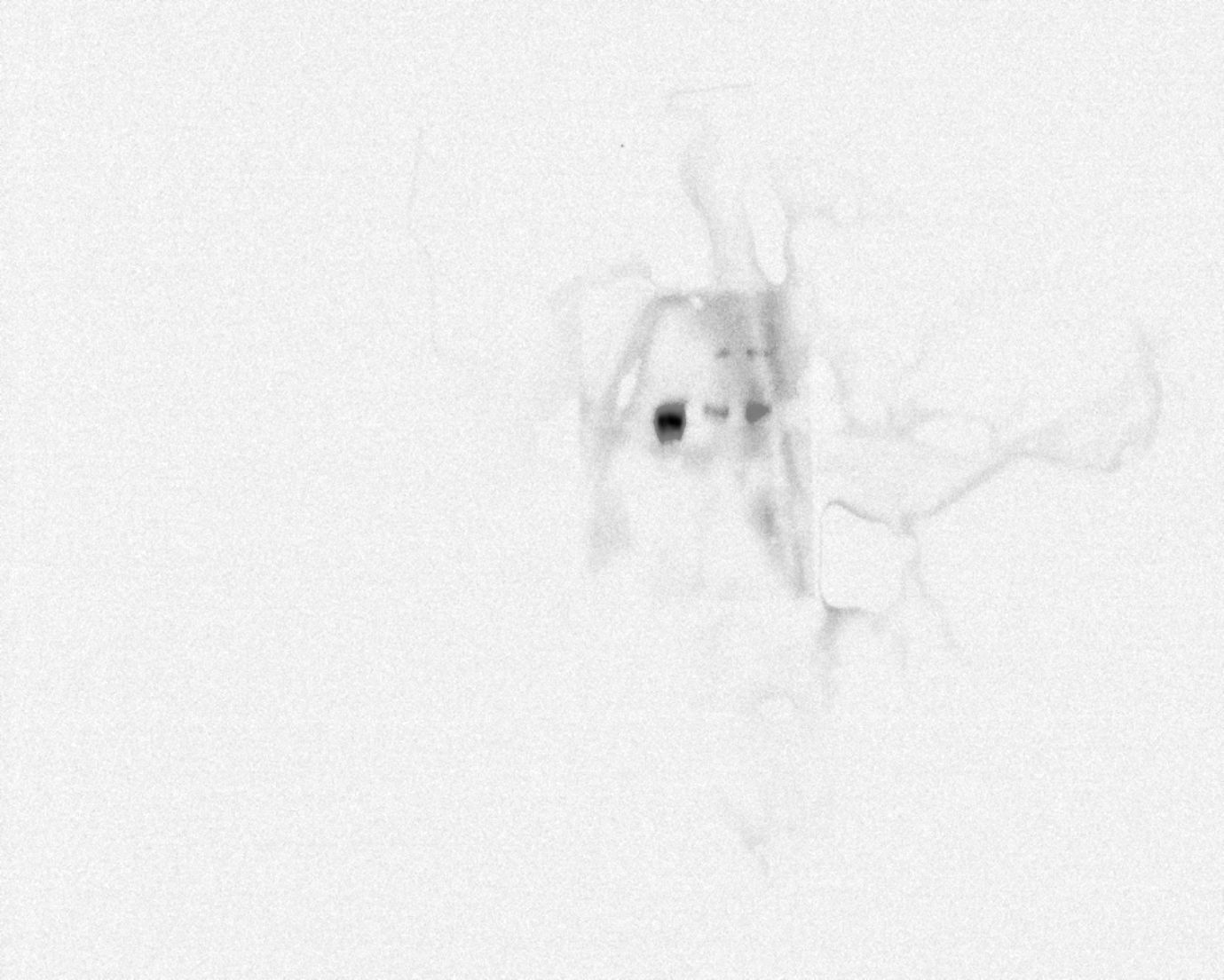

Supplement: Figure 4—source data 2. [file elife-102792-fig4-data2.zip › Figure 4-source data 2/Figure 4F_Source data 2/Figure 4F_Source data_Flag IP anti myc.tif]

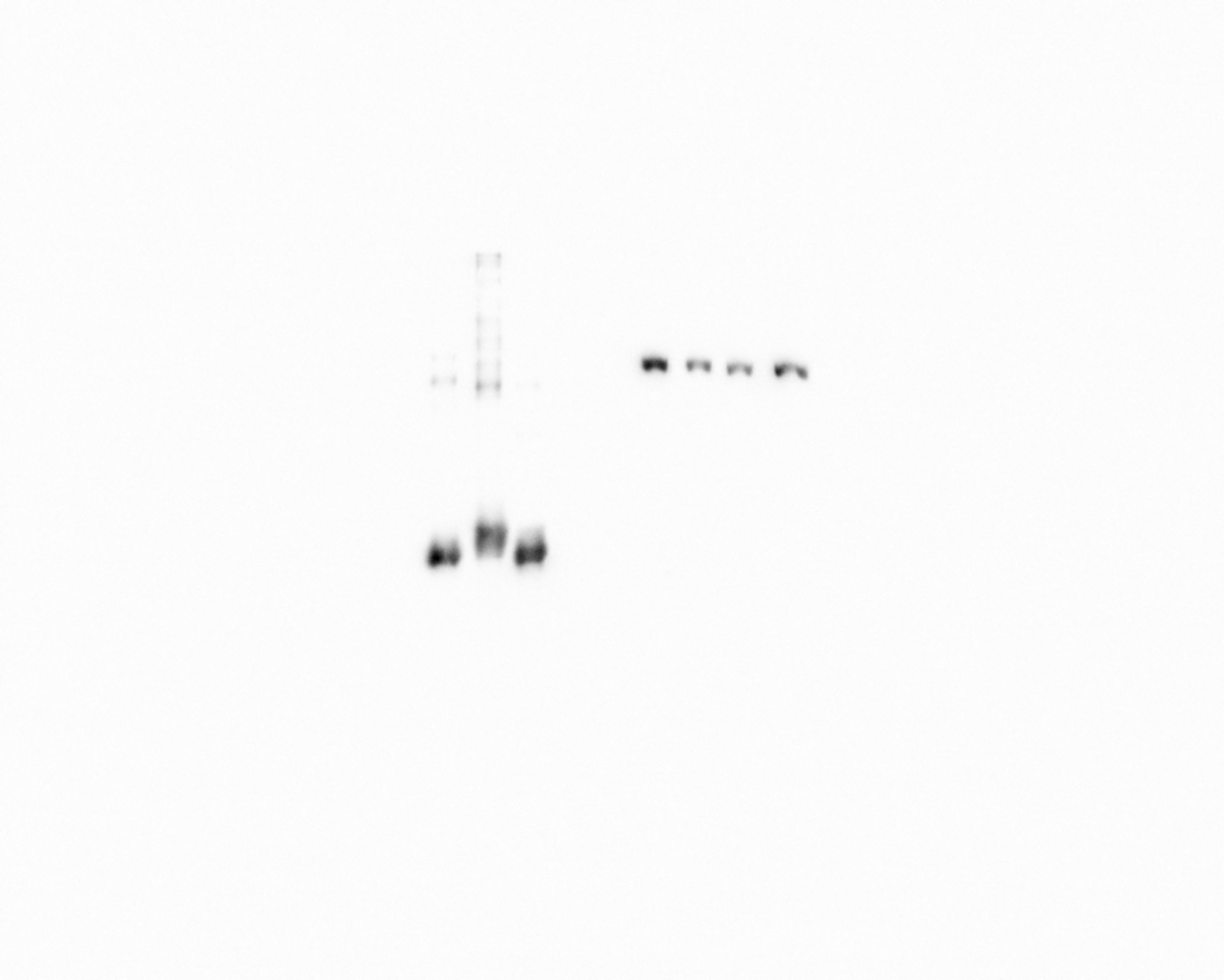

Supplement: Figure 4—source data 2. [file elife-102792-fig4-data2.zip › Figure 4-source data 2/Figure 4F_Source data 2/Figure 4F_Source data_Input anti myc GPN.tif]

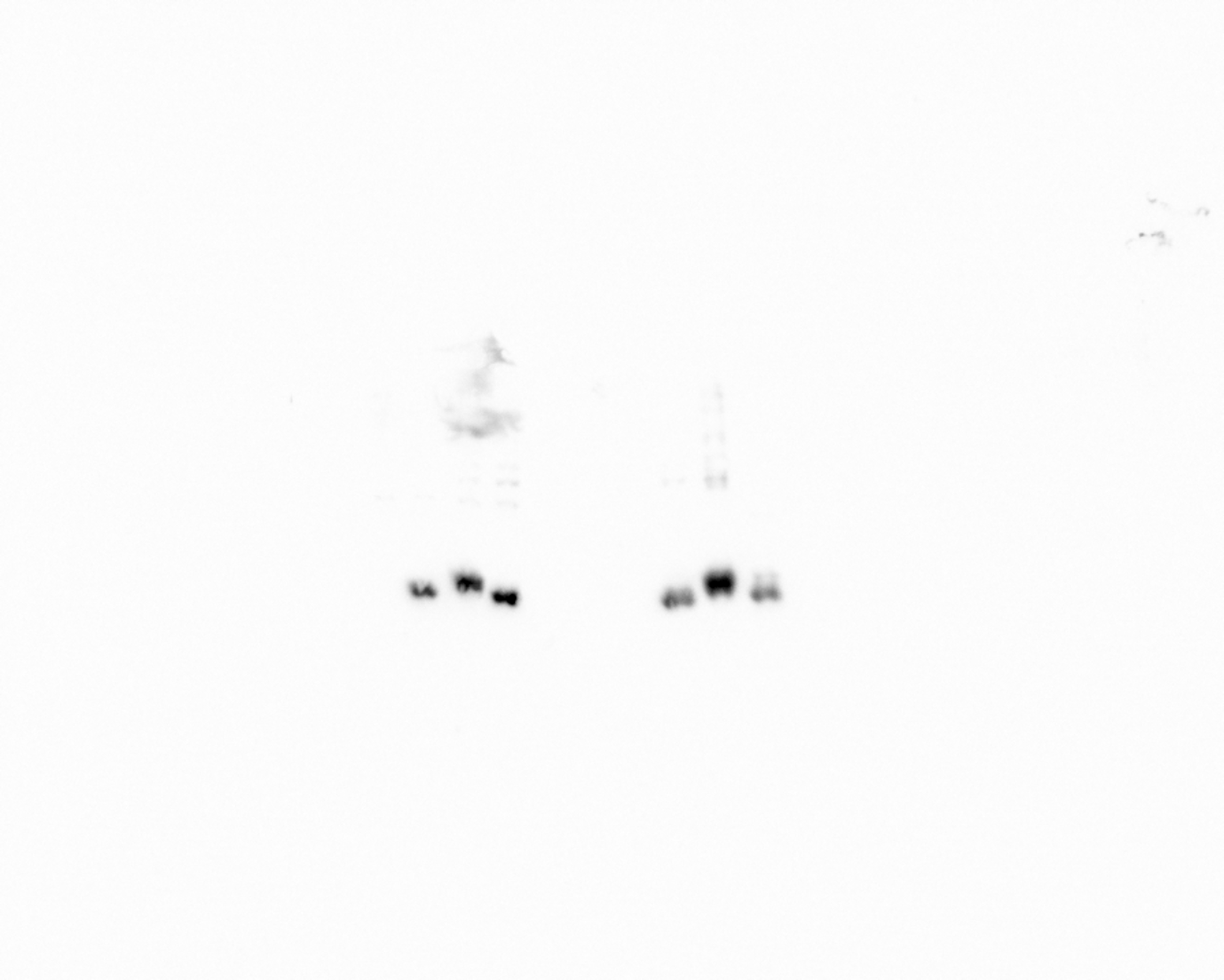

Supplement: Figure 4—source data 2. [file elife-102792-fig4-data2.zip › Figure 4-source data 2/Figure 4F_Source data 2/Figure 4F_Source data_Input IP-anti Flag .tif]

Figure 5G, Source Data

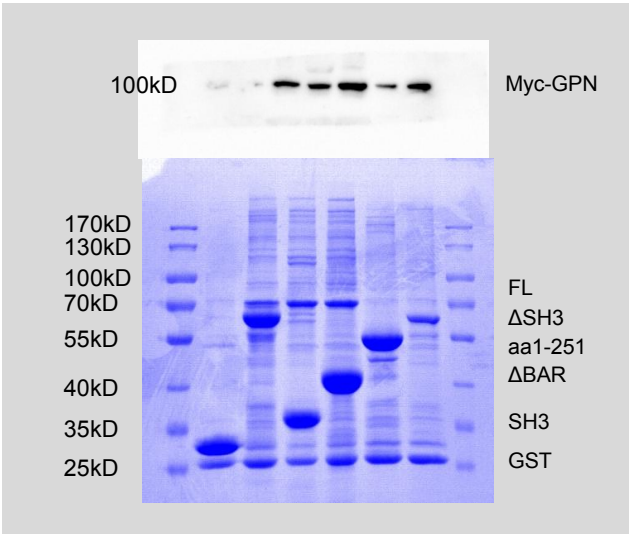

Supplement: Figure 5—source data 1. [file elife-102792-fig5-data1.zip › Figure 5-source data 1/Figure 5G_Source data 1.pdf]

Figure 5H, Source Data

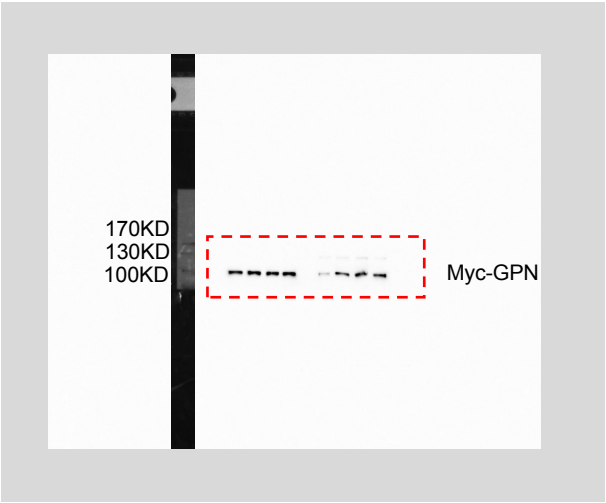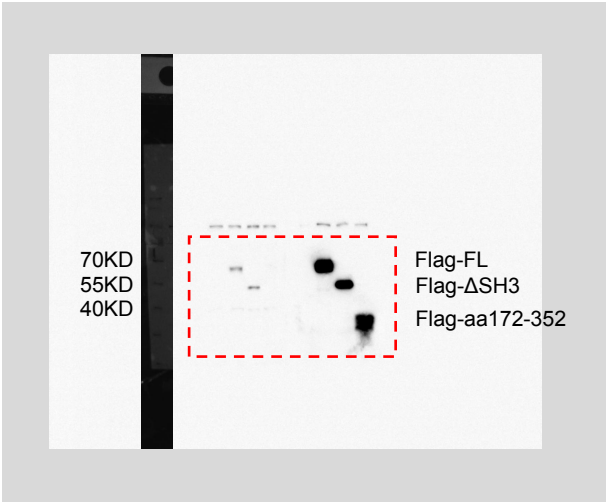

Supplement: Figure 5—source data 1. [file elife-102792-fig5-data1.zip › Figure 5-source data 1/Figure 5H_Source data 1.pdf]

Figure 5I, Source Data

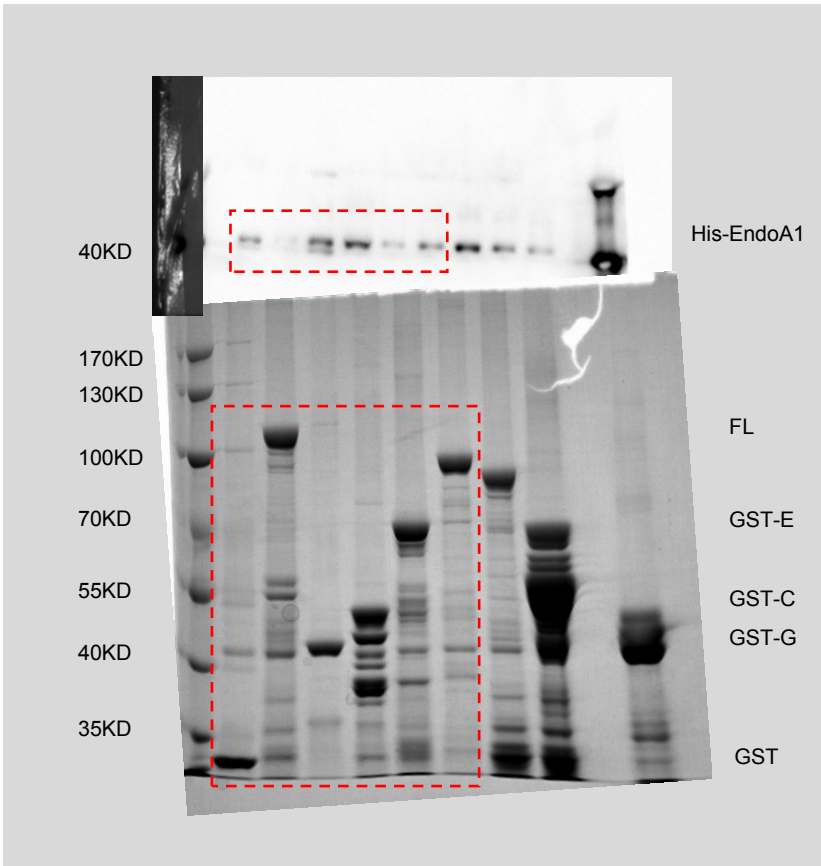

Supplement: Figure 5—source data 1. [file elife-102792-fig5-data1.zip › Figure 5-source data 1/Figure 5I_Source data 1.pdf]

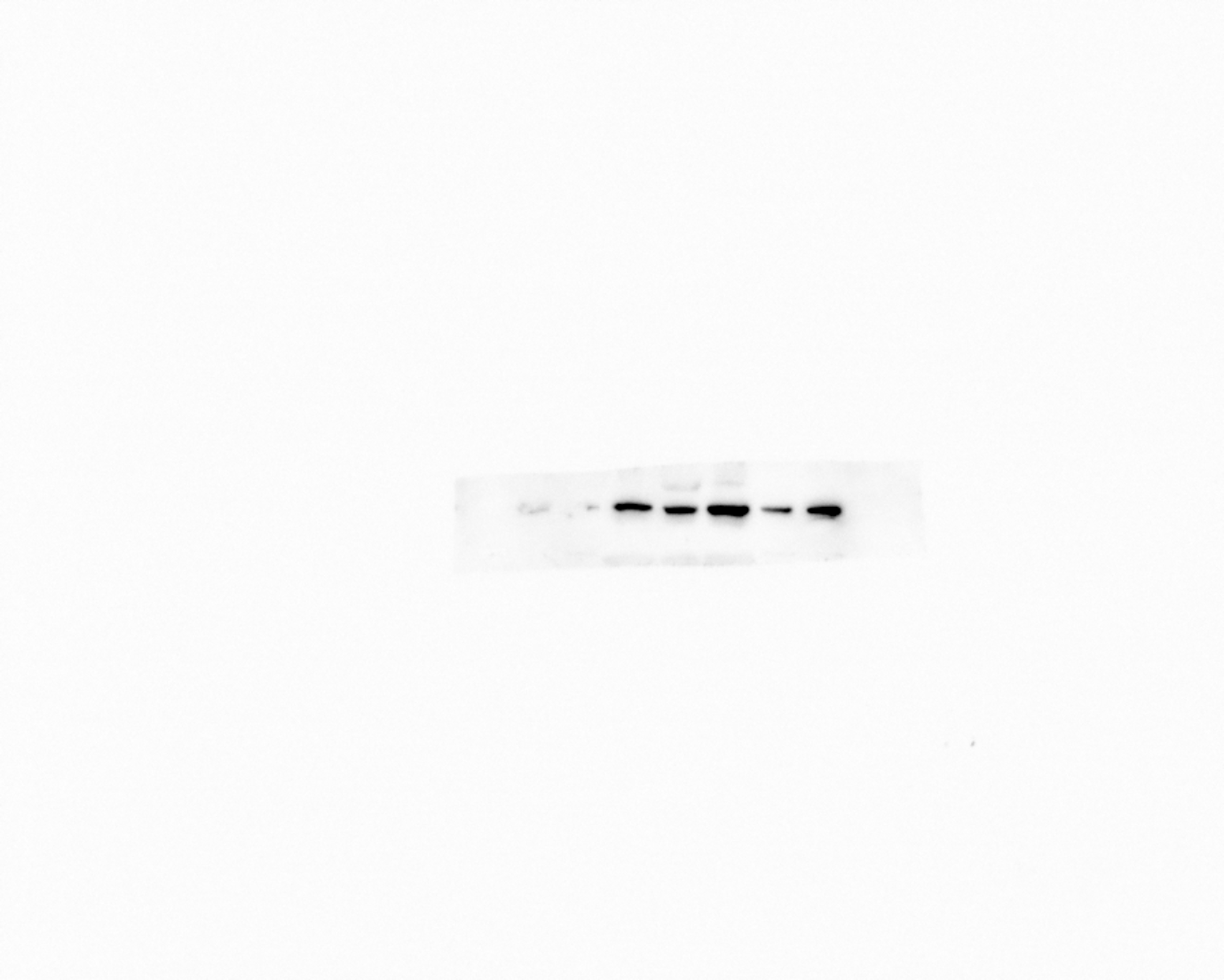

Supplement: Figure 5—source data 2. [file elife-102792-fig5-data2.zip › Figure 5-source data 2/Figure 5G_Source data2/Figure 5G_Source data_blot anti Myc-GPN.tif]

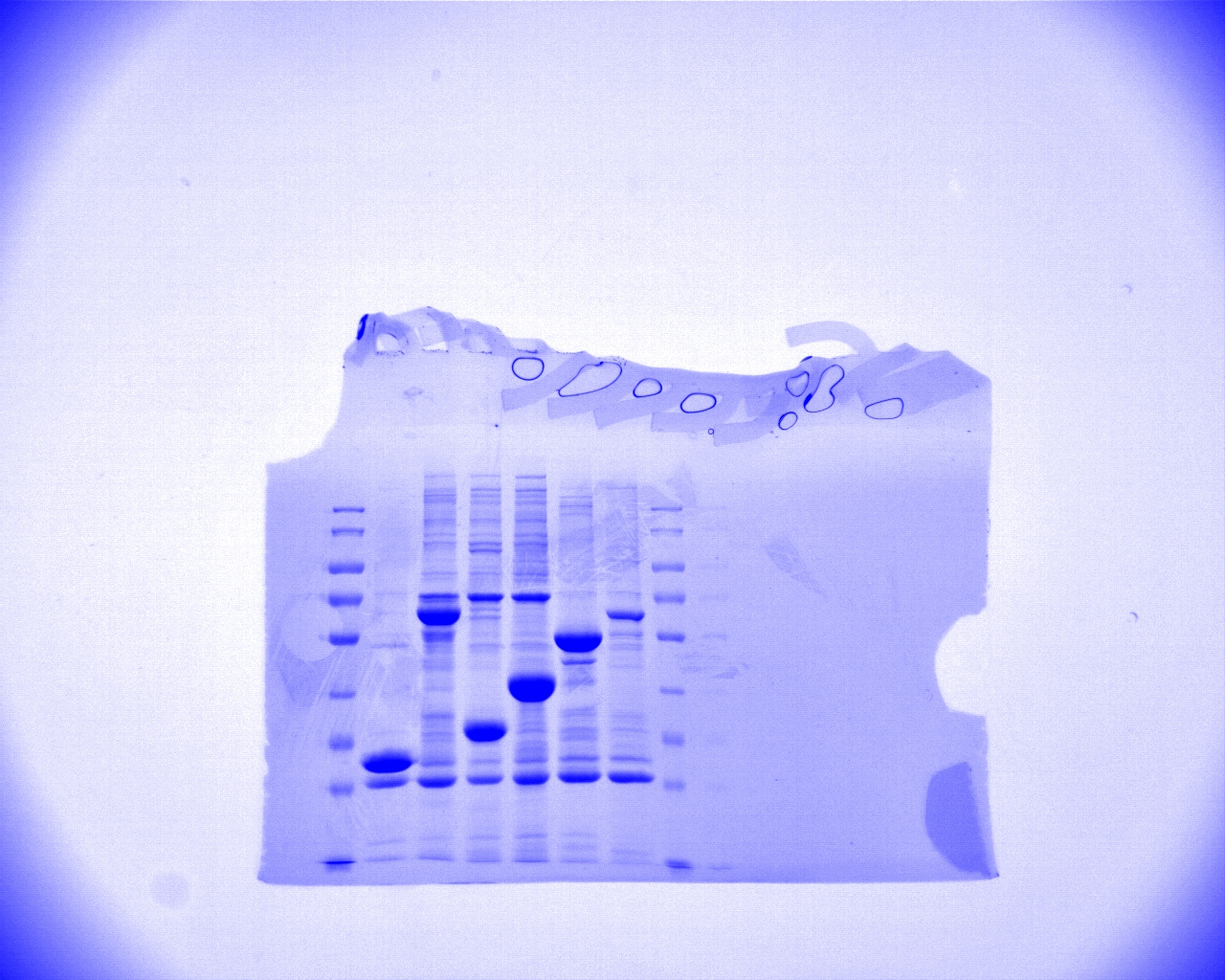

Supplement: Figure 5—source data 2. [file elife-102792-fig5-data2.zip › Figure 5-source data 2/Figure 5G_Source data2/Figure 5G_Source data_coomassie blue staining of GST-EndoA1.tif]

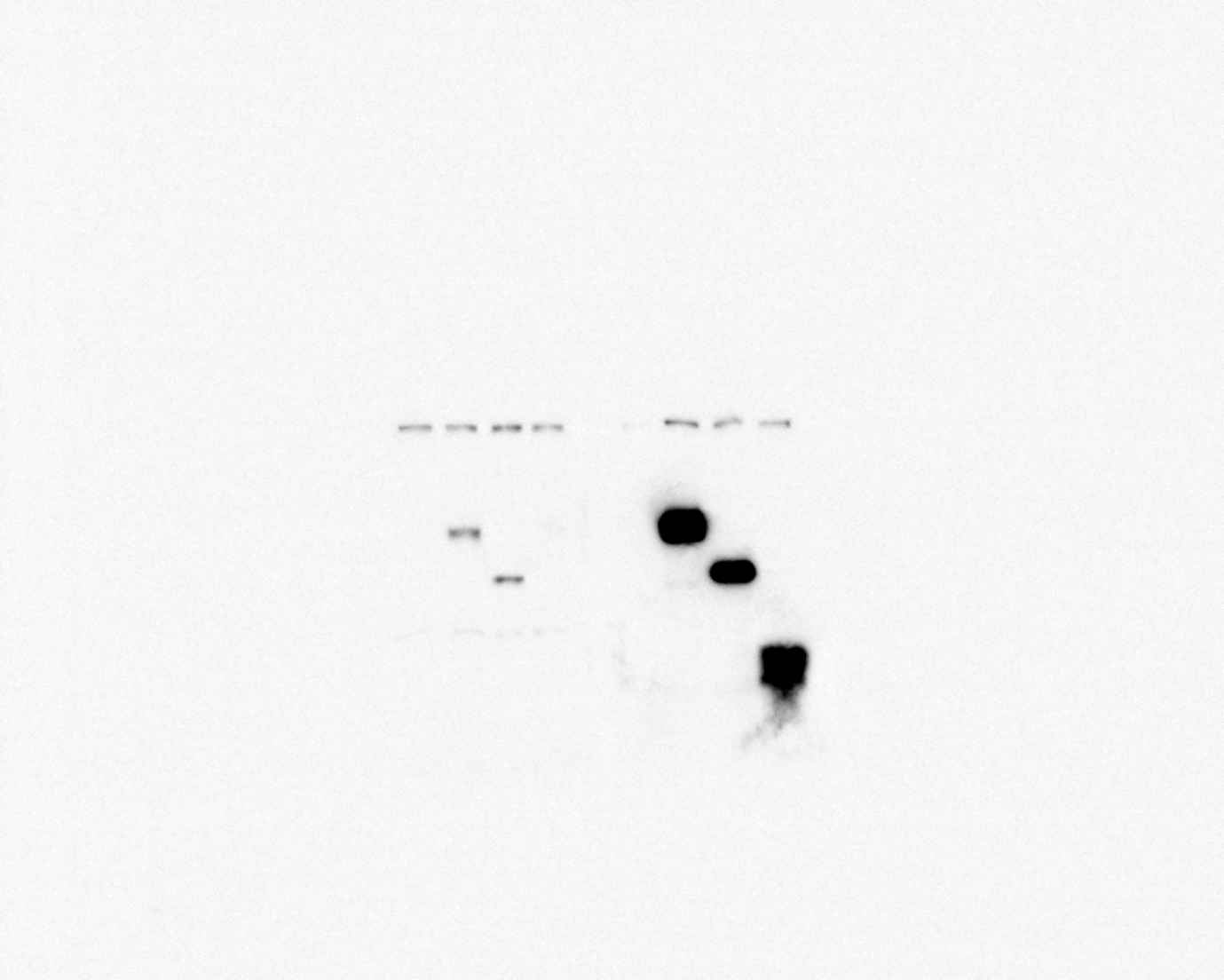

Supplement: Figure 5—source data 2. [file elife-102792-fig5-data2.zip › Figure 5-source data 2/Figure 5H_Source data 2/Figure 5H_Blot anti_flag-EndoA1.tif]

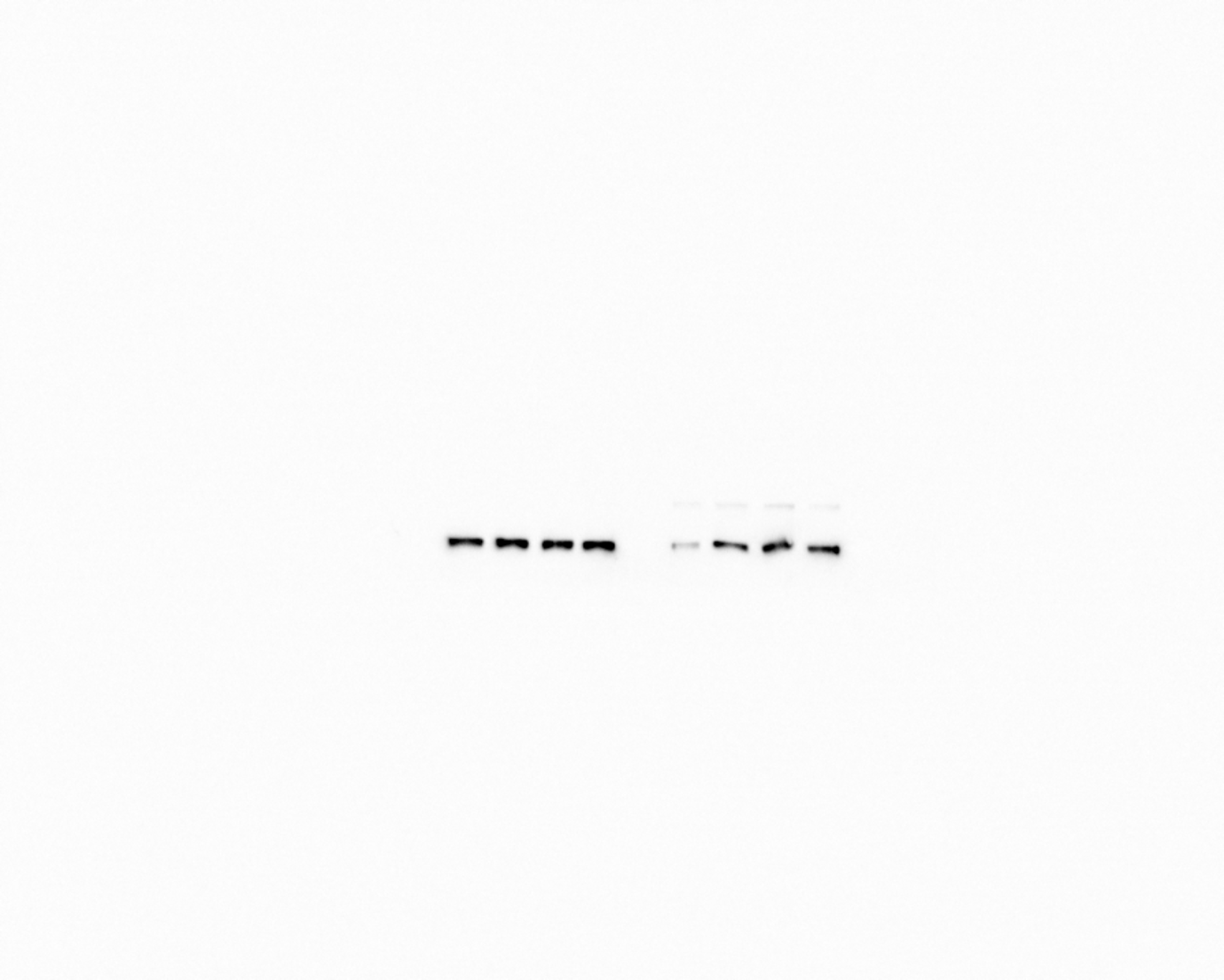

Supplement: Figure 5—source data 2. [file elife-102792-fig5-data2.zip › Figure 5-source data 2/Figure 5H_Source data 2/Figure 5H_Blot anti_myc-GPN.tif]

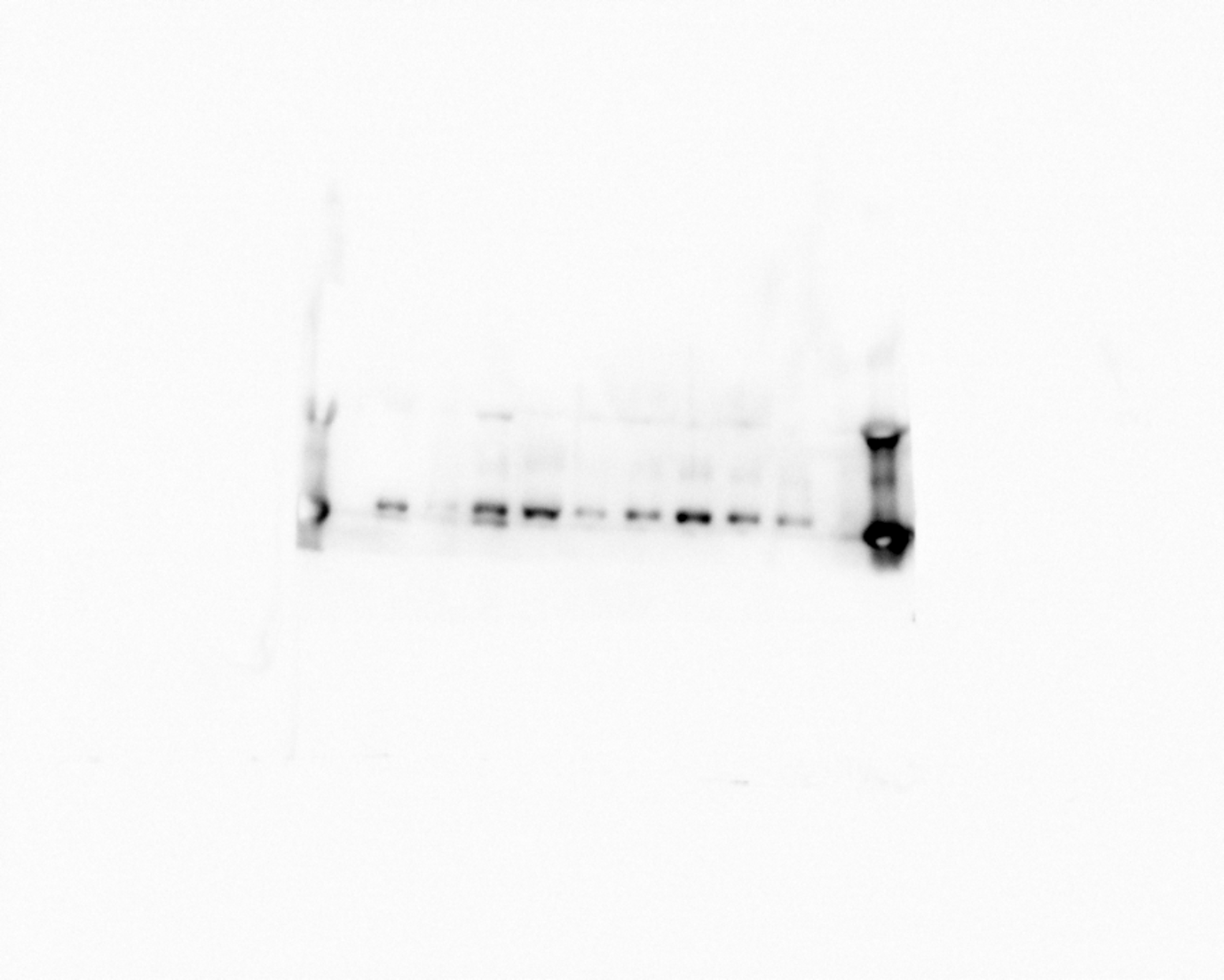

Supplement: Figure 5—source data 2. [file elife-102792-fig5-data2.zip › Figure 5-source data 2/Figure 5I_Source data 2/Figure 5I_anti His-EndoA1.tif]

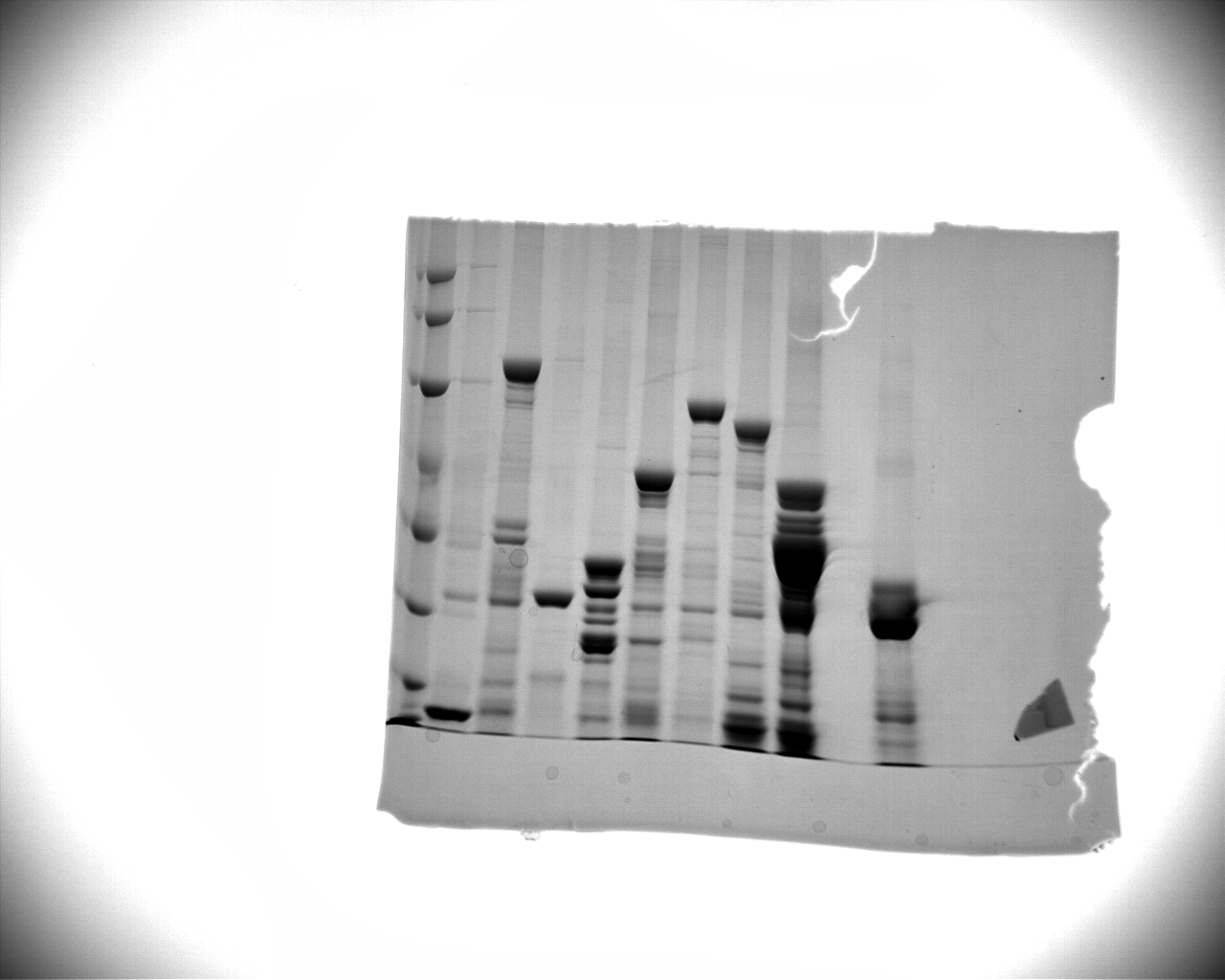

Supplement: Figure 5—source data 2. [file elife-102792-fig5-data2.zip › Figure 5-source data 2/Figure 5I_Source data 2/Figure 5I_Coomassie bule of GST GPN .tif.tif]
