# Supplementary material for: Endophilin A1 facilitates organization of the GABAergic postsynaptic machinery to maintain excitation-inhibition balance
Source: eLife. 2025 Oct 2;13:RP102792. doi: 10.7554/eLife.102792 (PMC12490859; doi:10.7554/eLife.102792)
Supplement: Source data 1. [file elife-102792-data1.docx]

**Primers used in this research**

| Constructs name | Primer sequence (5’-3’) |
| --- | --- |
| pCMV-Tag 3B-GPN F | agaggatctgagcccgggcggatccatggcgaccgagggaatgat |
| pCMV-Tag 3B-GPN R | tatcgataagcttgatatcgaattctcatagccgtccgatgacca |
| pGEX-4T-1-GPN FL F | aatcggatctggttccgcgtggatccatggcgaccgagggaatgat |
| pGEX-4T-1-GPN FL R | ggccgctcgagtcgacccgggaattctcatagccgtccgatgacca |
| pGEX-4T-1-GPN G (aa15-169) F | aatcggatctggttccgcgtggatccatccgtgtcggagtcctcac |
| pGEX-4T-1-GPN G (aa15-169) R | ggccgctcgagtcgacccgggaattctaaaaggtcaatggcatgag |
| pGEX-4T-1-GPN C (aa170-326) F | aatcggatctggttccgcgtggatcccgtgatgccattgtaaaagt |
| pGEX-4T-1-GPN C (170-326) R | ggccgctcgagtcgacccgggaattccatagacgtcagaggaaaag |
| pGEX-4T-1-GPN E (aa327-733) F | aatcggatctggttccgcgtggatccgacaaagccttcattacagt |
| pGEX-4T-1-GPN E (aa327-733) R | ggccgctcgagtcgacccgggaattcgatgaccatgacgtccacca |
| pGEX-4T-1 EEN1 ΔSH3 (Δaa295-346) | aatcggatctggttccgcgtggatcccaaatggatcagccttgc |
| pGEX-4T-1 EEN1 ΔSH3 (Δaa295-346) | ggccgctcgagtcgacccgggaattcctaatggggcagagcaaccag |
| pGEX-4T-1-EEN1ΔBAR (Δaa6-242) F | aatcggatctggttccgcgtggatccatgtcggtggcaggg |
| pGEX-4T-1-EEN1ΔBAR (Δaa6-242) R | ggccgctcgagtcgacccgggaattctgaagcttgtcttattct |
| Mycoplasma F： | TGCACCATCTGTCACTCTGTTAACCTC |
| Mycoplasma R | GGGAGCAAACAGGATTAGATACCCT |
